# *In Vitro* Viral Evolution Identifies a Critical Residue in the Alphaherpesvirus Fusion Glycoprotein B Ectodomain That Controls gH/gL-Independent Entry

**DOI:** 10.1128/mBio.00557-21

**Published:** 2021-05-04

**Authors:** Melina Vallbracht, Henriette Lötzsch, Barbara G. Klupp, Walter Fuchs, Benjamin Vollmer, Kay Grünewald, Marija Backovic, Felix A. Rey, Thomas C. Mettenleiter

**Affiliations:** a Institute of Molecular Virology and Cell Biology, Friedrich-Loeffler-Institut, Greifswald-Insel Riems, Germany; b Centre for Structural Systems Biology, Heinrich-Pette-Institut, Leibniz-Institut für Experimentelle Virologie, Hamburg, Germany; c Institut Pasteur, Structural Virology Unit, Department of Virology, CNRS UMR3569, Paris, France; Columbia University Medical College

**Keywords:** autonomous fusion, bovine herpesvirus, fusion regulation, gH/gL complex, glycoprotein B, herpesviruses, membrane fusion, pseudorabies virus, viral entry

## Abstract

Herpesvirus entry and spread requires fusion of viral and host cell membranes, which is mediated by the conserved surface glycoprotein B (gB). Upon activation, gB undergoes a major conformational change and transits from a metastable prefusion to a stable postfusion conformation. Although gB is a structural homolog of low-pH-triggered class III fusogens, its fusion activity depends strictly on the presence of the conserved regulatory gH/gL complex and nonconserved receptor binding proteins, which ensure that fusion occurs at the right time and space. How gB maintains its prefusion conformation and how gB fusogenicity is controlled remain poorly understood. Here, we report the isolation and characterization of a naturally selected pseudorabies virus (PrV) gB able to mediate efficient gH/gL-independent virus-cell and cell-cell fusion. We found that the control exerted on gB by the accompanying viral proteins is mediated via its cytosolic domain (CTD). Whereas gB variants lacking the CTD are inactive, a single mutation of a conserved asparagine residue in an alpha-helical motif of the ectodomain recently shown to be at the core of the gB prefusion trimer compensated for CTD absence and uncoupled gB from regulatory viral proteins, resulting in a hyperfusion phenotype. This phenotype was transferred to gB homologs from different alphaherpesvirus genera. Overall, our data propose a model in which the central helix acts as a molecular switch for the gB pre-to-postfusion transition by conveying the structural status of the endo- to the ectodomain, thereby governing their cross talk for fusion activation, providing a new paradigm for herpesvirus fusion regulation.

## INTRODUCTION

Enveloped viruses have evolved specialized surface glycoproteins, termed fusion proteins or viral fusogens, to catalyze the merger of viral and cell membranes for infectious entry and spread. To effect fusion, these proteins undergo a large-scale exothermic conformational change and refold from a high-energy metastable prefusion form to an energetically more favorable postfusion conformation (1). The different molecular architectures of fusion proteins led to the identification of three distinct structural classes (I to III) (2). According to the trigger of the fusogenic transition, viral fusion proteins can be divided into two major categories: those that are activated by binding of protons in the acidic environment of the endosome or those triggered by interaction with one or more specific receptors at the cell surface at neutral pH. The second category requires additional control proteins to ensure that fusion takes place at the right time and space.

Herpesviruses are large, enveloped double-stranded DNA viruses. Members of the *Herpesviridae* can enter and infect many vertebrate species using a complex multicomponent fusion machinery (3). Envelope glycoprotein B (gB) is their conserved *bona fide* fusion protein. Based on the crystal structure of its stable, trimeric postfusion state, which is available for five different herpesviruses, including the alphaherpesviruses pseudorabies virus (PrV; *Suid alphaherpesvirus 1*) (4, 5), human herpes simplex virus 1 (HSV-1) (6), and varicella-zoster virus (VZV) (7), gB was identified as a class III viral fusion protein. Typically, class III fusogens, which include the G protein of rhabdoviruses, baculovirus glycoprotein 64 (gp64), and thogotovirus Gp, mediate both receptor binding and autonomous low-pH-triggered membrane fusion (2). In stark contrast, herpesvirus gB is not an autonomous fusogen but requires activation by accompanying proteins to drive pH-independent membrane fusion at the plasma membrane (8). The current model for alphaherpesvirus entry proposes that receptor-engagement by the subfamily-specific receptor-binding protein gD initiates a cascade of events in which a structural change in gD signals to the conserved complex of membrane bound gH with anchorless gL (gH/gL), which in turn triggers the gB fusogenic conformational change (3, 9–11). The forces maintaining gB in its metastable prefusion conformation and how they are released to execute fusion remain largely unknown.

Functional studies on alphaherpesviruses indicate that the very large and structured gB cytoplasmic domain (CTD) (90 amino acids [aa] for PrV gB) (12), which lacks an equivalent among the short and unstructured cytosolic tails of low-pH-triggered class III fusion proteins (see Fig. S1 in the supplemental material), is crucial for its fusogenicity. While removal of the entire CTD renders gB nonfunctional (13, 14), truncations of, or point mutations within the gB CTD resulted in increased fusion activity, pointing towards the gB CTD as a key regulatory element that negatively controls gB fusogenicity (13, 15–20). Multiple studies have shown that in the absence of the CTD, the gB ectodomain (4) spontaneously adopts the stable postfusion conformation (4, 6, 7, 21, 22), implying that the membrane anchor, the CTD, and/or gH/gL is involved in maintaining gB in its fusion-active, metastable prefusion state (3). Indeed, recent structural studies of full-length gB on extracellular vesicles (HSV-1) (23) and intact human cytomegalovirus (HCMV) virions (24) allowed elucidation of a putative prefusion structure, albeit to limited resolution, supporting the hypothesis that the CTD and accompanying viral proteins are important to maintain gB in a fusion-active state. Nevertheless, introduction of a mutation stabilizing the prefusion state of HSV-1 gB was required to allow its determination to subnanometer resolution and the identification of the arrangements of all subdomains (25).

10.1128/mBio.00557-21.1 FIG S1 Comparison of cytoplasmic domains of class III fusion proteins. (A) Schematic map of class III fusion proteins and alignment of amino acid sequences of the cytoplasmic domains (CTDs) of the rhabdovirus vesicular stomatitis virus (VSV) G protein, baculovirus Autographa californica nuclear polyhedrosis virus (AcNMPV) gp64, Thogotovirus (THOV) and Dhori virus (DOHV) Gps, and herpesvirus gBs, including those of pseudorabies virus (PrV), avian infectious laryngotracheitis virus (ILTV), bovine alphaherpesvirus-1 (BoHV-1), and herpes simplex virus 1 (HSV-1). Two alpha-helical domains in the CTDs of PrV, ILTV, and BoHV-1 gB were predicted by JPred4 and are highlighted in yellow and orange. Amino acids forming HSV-1 helices h1a, h1b, h2, and h3 are indicated in blue, yellow, and orange, respectively. Amino acids boxed in red and highlighted by a red asterisk indicate the position at which an artificial stop codon was introduced for generation of C-terminally truncated gB variants. (B) Ribbon diagram of the trimeric HSV-1 gB CTD and transmembrane domain (TMD) (PDB 5V2S). The CTD alpha-helices h1a, h1b, h2, and h3 of the three protomers are labeled and colored as in panel A. Download FIG S1, TIF file, 1.7 MB. Copyright © 2021 Vallbracht et al. 2021 Vallbracht et al. https://creativecommons.org/licenses/by/4.0/ This content is distributed under the terms of the Creative Commons Attribution 4.0 International license.

In this study, we further investigated the functional relevance of the gB CTD during virus infection using reversion analysis, i.e., *in vitro* evolution of a PrV mutant expressing a C-terminally truncated gB variant lacking the 60 C-terminal amino acids (PrV-gB^ΔCTD2^) (26). Although PrV-gB^ΔCTD2^ is deficient in entry, it is still able to spread from cell to cell (13, 26). This exceptional phenotype was used for serial *in vitro* passaging to select for mutations restoring viral infectivity. Reversion analysis is a powerful tool to select for mutations compensating a strong defect in the parental virus, assisting in the identification of significant structural and functional inter- and intramolecular relationships, which are poorly accessible via direct rational approaches (3, 26–33). Considering the proposed role of the gB CTD in maintaining the prefusion conformation, we hypothesized that compensatory mutations acquired by infectious revertants would reveal functionally important sites, including residues affecting the stability of prefusion gB.

Using this approach, we identified a PrV gB variant expressed by an infectious revertant that is hyperfusogenic and able to mediate autonomous *in vitro* cell-cell fusion in a gH/gL-independent manner, representing a hitherto undescribed phenotype. In-depth characterization at sequence, functional, and comparative structural levels allowed us to define a short conserved alpha-helix in domain V of the gB ectodomain as a central switch that is decisive for the gB pre-to-postfusion transition. A single point mutation of a conserved asparagine residue in this regulatory helix compensated for absence of the gB CTD and conferred gH/gL independence during viral entry and cell-cell spread. Strikingly, the same mutation was able to transform gB of the closely related *Varicellovirus Bovine alphaherpesvirus 1* (BoHV-1) but also gB of the distantly related *Iltovirus* avian infectious laryngotracheitis virus (ILTV; *Gallid alphaherpesvirus 1*) into gH/gL-independent fusion proteins, arguing for a common mechanism among alphaherpesvirus gB. Overall, our findings support a model in which the conformational change of the alphaherpesvirus gB ectodomain for fusion is under the allosteric control of the gB endodomain and that this cross talk is governed by a newly discovered regulatory element that eventually controls the fusogenic conformational change of gB.

## RESULTS

### Isolation of infectious PrV-gB^ΔCTD2^Pass.

**FIG 1:**
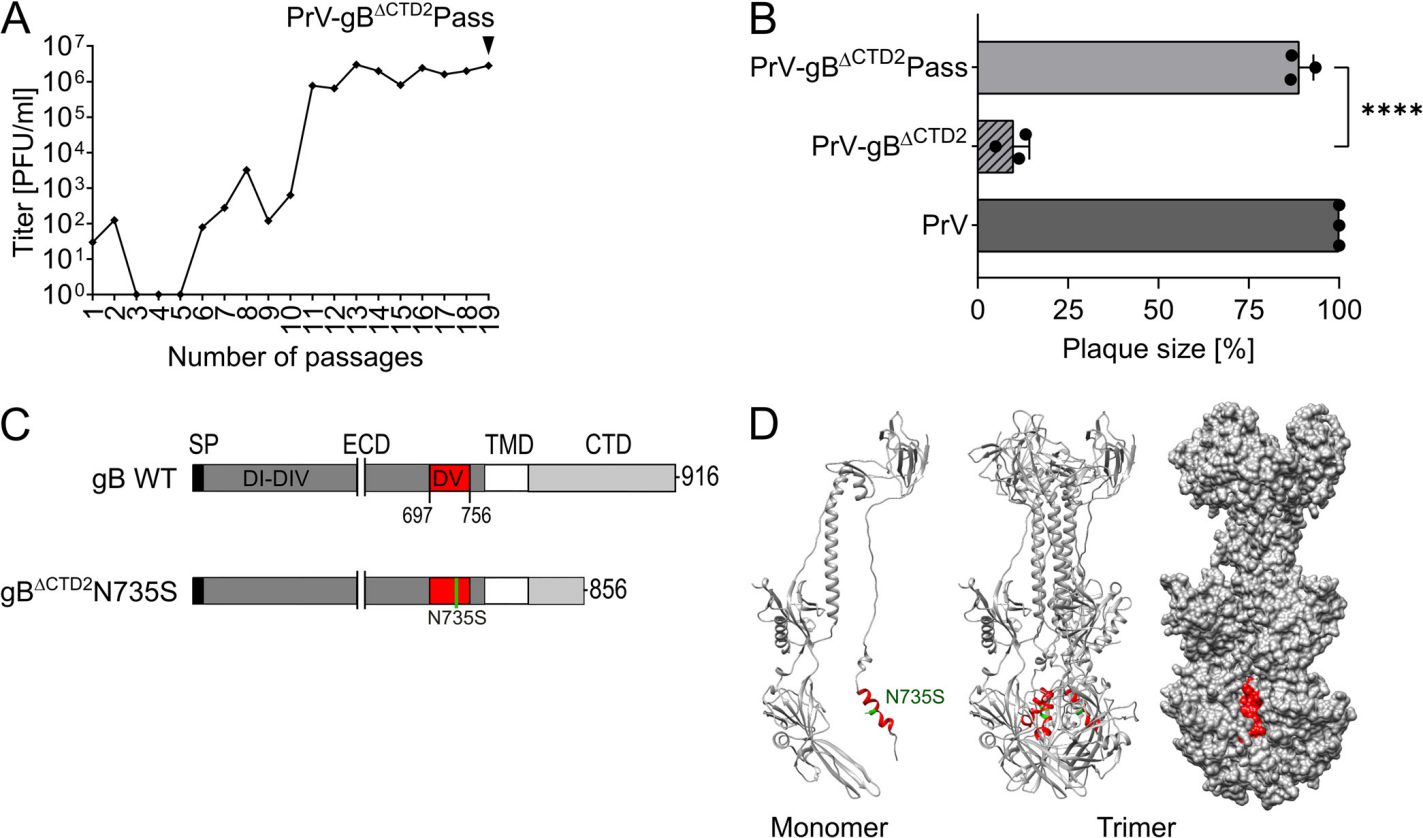
Reversion analysis of PrV-gB^ΔCTD2^. (A) PrV-gB^ΔCTD2^ expressing gB lacking the 60 C-terminal amino acids was passaged in RK13 cells by repeated coseeding of infected and noninfected cells. Supernatant titers of each passage were determined on RK13 cells and are given in PFU per milliliter. Infectious revertant virus PrV-gB^ΔCTD2^Pass was harvested from supernatant of the 19th passage (black arrowhead). (B) Plaque sizes of PrV, nonpassaged but wild-type gB-complemented PrV-gB^ΔCTD2^, and revertant PrV-gB^ΔCTD2^Pass were determined 48 h postinfection of RK13 cells. Shown are mean relative plaque areas expressed as percentage of WT PrV and corresponding standard deviations (*n* = 3). Two-tailed Welch’s *t* test; ****, *P* < 0.001. (C) Schematic diagram of PrV gB wild-type (WT), gB^ΔCTD2^, and gB^ΔCTD2^N735S. gB domain (D) V (residues 697 to 756) is highlighted in red, and the N735S mutation in green. SP, signal peptide; ECD, ectodomain; TMD, transmembrane domain; CTD, cytoplasmic domain. (D) Ribbon diagram of the PrV gB ectodomain monomer (left) and trimer (middle), with surface representation (right). DV helix (residues 728 to 742) is depicted in red, and N735 is marked in green; PDB 6ESC. Diagram was made using UCSF Chimera (62).

PrV-gB^ΔCTD2^ expressing gB lacking the 60 C-terminal amino acids (gB^ΔCTD2^) (Fig. 1C) was passaged in rabbit kidney (RK13) cells, and an infectious revertant virus termed PrV-gB^ΔCTD2^Pass was isolated by plaque purification of the 19th passage (Fig. 1A). The cell-cell spread ability of PrV-gB^ΔCTD2^Pass was investigated 48 h after infection of RK13 cells (Fig. 1B). In comparison to nonpassaged PrV-gB^ΔCTD2^ (∼10% of PrV wild type [WT] plaque size), PrV-gB^ΔCTD2^Pass formed significantly larger plaques, reaching approximately 90% of the WT virus plaque size (Fig. 1B).

### PrV-gB^ΔCTD2^Pass had acquired a single compensatory N735S mutation in the gB ectodomain.

Viral DNA from the infectious PrV-gB^ΔCTD2^Pass plaque isolate was analyzed for mutations in the genes encoding the fusion-associated envelope glycoproteins gD (US6), gH (UL22), gL (UL1), and gB (UL27). Only a single point mutation in the gB gene was acquired during passaging, leading to an exchange of asparagine (N) at position 735 to serine (S) (Fig. 1C and D), while no alterations were found in the other coding regions tested. Mapping of N735 into the trimeric postfusion structure of the PrV gB ectodomain revealed its location in a short alpha-helix within domain (D) V, where it is located on the outside of the trimer nested between the interfaces formed by the other two protomers (Fig. 1D). For further functional investigations, the complete gB open reading frame was amplified from genomic PrV-gB^ΔCTD2^Pass DNA and cloned into the eukaryotic expression vector pcDNA3.

### N735S imparts hyperfusogenic gH/gL-independent cell-cell fusion.

**FIG 2:**
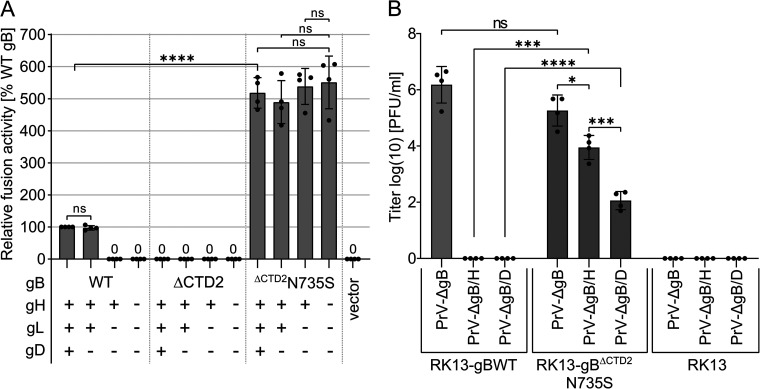
gB^ΔCTD2^N735S drives strong autonomous cell-cell fusion and supports gH/gL-independent virus entry. (A) *In vitro* fusion activity of PrV WT gB, gB^ΔCTD2^, and gB^ΔCTD2^N735S in different combinations with WT gH, gL, and gD was determined 18 h after transfection of RK13 cells. Results from fusion assays with WT gB, gH, gL, and gD were set as 100%, and empty vector served as a negative control. Mean relative values and corresponding standard deviations are shown (*n* = 4). Mean values below 50% are given as numbers. (B) Noncomplementing RK13 cells or RK13-gBWT and RK13-gB^ΔCTD2^N735S cell lines were infected with phenotypically complemented PrV-ΔgB, PrV-ΔgB/H, or PrV-ΔgB/D at an MOI of 3. Twenty-four hours postinfection (p.i.), progeny virus was harvested and titrated on RK13-gBWT cells. Mean titers in PFU per milliliter and corresponding standard deviations are shown (*n* = 4). (A and B) Two-tailed Welch’s *t* test; ns, not significant; *, *P* < 0.05; ***, *P* < 0.001; ****, *P* < 0.0001.

To investigate the impact of the N735S mutation on gB fusogenicity, gB^ΔCTD2^N735S was tested in a transfection-based cell-cell fusion assay (34) (Fig. 2A). RK13 cells were transfected with expression plasmids encoding PrV WT gB, gB^ΔCTD2^, or gB^ΔCTD2^N735S. Optionally, expression plasmids encoding PrV WT gH, gL, and gD were cotransfected (15, 30). Fusion activities were determined 18 h posttransfection by multiplication of the mean area of syncytia containing three or more nuclei by the number of syncytia. Fusion activities with the four WT glycoproteins served as a positive control and were set as 100%, while fusion assays with empty vector pcDNA3 served as a negative control. As expected, coexpression of PrV WT gB, gH, and gL resulted in the formation of multiple syncytia, whereas omission of gH/gL completely abolished fusion. PrV gD was not required for cell-cell fusion as reported earlier (15, 30) (Fig. 2A). No syncytium formation was detectable in assays with C-terminally truncated gB^ΔCTD2^, supporting the notion that the PrV gB CTD plays a crucial role for gB fusogenicity. Surprisingly, the N735S mutation not only restored but strongly enhanced the fusion activity of gB^ΔCTD2^ compared to that of WT gB. This hyperfusion phenotype of gB^ΔCTD2^N735S was observed independent of the presence of gH and/or gL (Fig. 2A). Overall, these findings demonstrate that the N735S mutation not only compensates for the absence of the C-terminal 60 aa of the gB CTD but also for absence of gH/gL. A hyperfusogenic gH/gL-independent phenotype as described here for PrV gB^ΔCTD2^N735S has never been reported for any herpesvirus gB.

### gB^ΔCTD2^N735S supports gH/gL-independent viral entry.

To investigate whether gB^ΔCTD2^N735S is also able to drive gH/gL- or gD-independent viral entry, virus mutants simultaneously lacking the gB and gH (PrV-ΔgB/H), or gB and gD genes (PrV-ΔgB/D) (35), were complemented in *trans* with gB^ΔCTD2^N735S and used for infection experiments (Fig. 2B). Since cellular expression of gB^ΔCTD2^N735S led to a rapid induction of strong cell-cell fusion, we generated RK13 cell lines stably expressing PrV gB^ΔCTD2^N735S or PrV gB WT under the control of the native gB (UL27) promoter, allowing expression only upon transactivation by PrV infection. The resulting RK13-gB^ΔCTD2^N735S and RK13-gBWT cells were infected with phenotypically complemented PrV-ΔgB/H, PrV-ΔgB/D, or with a PrV mutant lacking only the gB gene (PrV-ΔgB) (13) at a multiplicity of infection (MOI) of 3. Progeny virus was harvested 24 h postinfection and titrated on RK13-gBWT cells (Fig. 2B). Progeny virus was also used for Western blot analyses to verify correct glycoprotein expression of the different PrV mutants (see Fig. S2).

10.1128/mBio.00557-21.2 FIG S2 Western blot analyses of PrV deletion mutants. PrV mutants lacking the genes encoding gB (PrV-ΔgB), gB and gH (PrV-ΔgB/H), or gB and gD (PrV-ΔgB/D) were propagated on noncomplementing RK13 cells or RK13 cells stably expressing gB^ΔCTD2^N735S or PrV WT gB under control of the native gB promoter. Lysates of infected cells were used for Western blot analyses. Expression of PrV gB, gD, and gH was detected using polyclonal rabbit antisera as indicated and peroxidase-conjugated secondary antibodies. Full-length PrV gBa is cleaved by cellular furin resulting in gBb and gBc. Molecular masses (kDa) of marker proteins are given. Download FIG S2, TIF file, 0.9 MB. Copyright © 2021 Vallbracht et al. 2021 Vallbracht et al. https://creativecommons.org/licenses/by/4.0/ This content is distributed under the terms of the Creative Commons Attribution 4.0 International license.

PrV WT gB efficiently complemented PrV-ΔgB to titers of 10^5^ PFU/ml but was not able to support entry of PrV-ΔgB/H or PrV-ΔgB/D (Fig. 2B). gB^ΔCTD2^N735S was able to complement PrV-ΔgB, and final titers did not significantly differ from those obtained for PrV-ΔgB complemented with WT gB. Remarkably, gB^ΔCTD2^N735S was also able to support gH/gL-independent entry by PrV-ΔgB/H, reaching titers of ∼10^4^ PFU/ml as well as entry of PrV-ΔgB/D to titers of ∼10^2^ PFU/ml. These results demonstrate that gB^ΔCTD2^N735S not only mediates highly efficient autonomous *in vitro* membrane fusion but also a significant level of gH/gL-independent entry. Although gH/gL is apparently not required to trigger and/or stabilize gB^ΔCTD2^N735S for fusion during entry, receptor engagement by gD augments viral entry presumably by providing a stable platform for action of the fusogen.

### N735S and the physical length of the gB CTD dictate gH/gL dependence.

**FIG 3:**
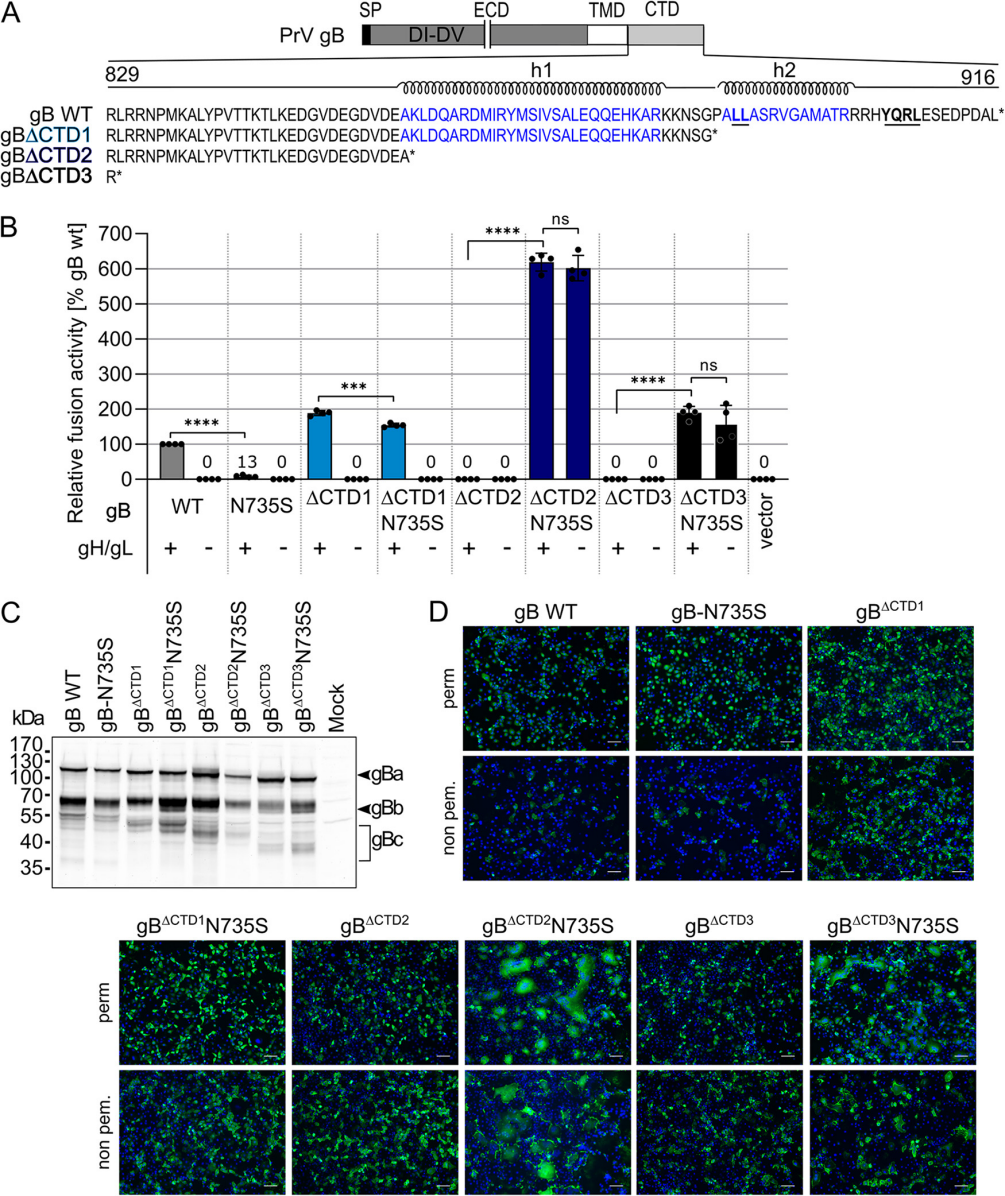
gB cytoplasmic domain dictates gH/gL dependence. (A) Schematic diagram of PrV gB and amino acid sequence of the predicted cytoplasmic domain (CTD) of PrV gB WT and gB mutants with truncations of the 29 or 60 C-terminal amino acids or complete removal of the CTD. Predicted alpha-helices h1 and h2 (JPred4 [36]) are indicated, and two functional endocytosis motifs are highlighted in bold and are underlined. SP, signal peptide, ECD, ectodomain; TMD, transmembrane domain. (B) Fusion activities of the indicated gB variants with and without gH/gL were determined 18 h after transfection of RK13 cells with corresponding expression plasmids. RK13 cells transfected with empty vector served as a negative control. Fusion activity obtained for WT gB, gH, and gL was set as 100%, and mean relative values and corresponding standard deviations are shown (*n* = 4). Mean values below 50% are indicated by numbers. Two-tailed Welch’s *t* test; ns, not significant; ***, *P* < 0.001; ****, *P* < 0.0001. (C) Western blot analyses of PrV gB variants 18 h after transfection of RK13 cells. PrV gB was detected using a specific rabbit antiserum and peroxidase-conjugated secondary antibody. Signals of uncleaved gBa or furin-cleaved subunits gBb and gBc are marked, and the molecular masses of marker proteins are indicated. (D) Total and surface expression of the gB variants analyzed by indirect immunofluorescence 18 h posttransfection of RK13 cells using a Leica DMi8 microscope (Leica Microsystems, Wetzlar, Germany); PrV gB was detected using PrV gB-specific rabbit antiserum and Alexa Fluor 488-conjugated secondary antibodies (green). Nuclei were counterstained with Hoechst 33342 (blue). Bars, 100 nm.

We next investigated whether substitution of N735 by serine in PrV WT gB or gB derivatives with different truncations of the CTD would allow for gH/gL-independent membrane fusion. The PrV gB CTD contains two predicted alpha-helical domains (h1 and h2; JPred4 [36]) (Fig. 3A; Fig. S1). As inferred from the predictions and comparative sequence-structure analysis with the gB CTD of HSV-1 gB (12), putative PrV gB h1 and h2 might exert similar functions as HSV-1 gB h2 and h3, whereby a major role in membrane binding was attributed to h3 (12, 20) (Fig. S1). The N735S mutation was inserted into PrV WT gB and gB variants lacking the 29 C-terminal amino acids, including h2 (gB^ΔCTD1^), or the complete CTD (gB^ΔCTD3^) (Fig. 3A). Fusion activities of these mutants were tested in assays with and without gH/gL (Fig. 3B).

Removal of the complete PrV gB CTD (PrV gB^ΔCTD3^) resulted in a loss of function, whereas expression of PrV gB^ΔCTD1^ led to enhanced but gH/gL-dependent fusion activity (Fig. 3B). Unexpectedly, introduction of the N735S mutation into full-length gB (gB-N735S) and hyperfusogenic gB^ΔCTD1^ (gB^ΔCTD1^N735S) led to significantly decreased fusion levels in the presence of gH/gL. In contrast, introduction of the N735S mutation into gB^ΔCTD3^ (gB^ΔCTD3^N735S) rescued fusion activity, and fusion levels reached approximately 200% compared to that of PrV WT glycoproteins. As observed for gB^ΔCTD2^N735S, gB^ΔCTD3^N735S was able to promote membrane fusion in the absence of gH/gL. In contrast, neither gB-N735S nor gB^ΔCTD1^N735S was functional in the absence of gH/gL, indicating that both the N735S mutation and the absence of the alpha-helical motifs in the CTD contribute to gH/gL-independent gB activation. These results indicate that the control exerted on gB by gH/gL is mediated via the gB CTD and that appropriate truncation or complete absence of the gB CTD is a prerequisite for efficient gH/gL-independent fusion by gB carrying the N735S mutation.

To exclude a possible influence of the N735S mutation on gB expression or processing, the same panel of gB mutants was investigated by Western blotting and indirect immunofluorescence analyses of transfected RK13 cells (Fig. 3C to E). PrV gB processing involves proteolytic cleavage behind ^501^RRARR^505^ by cellular furin in the Golgi apparatus, resulting in two disulfide-bonded subunits, gBb (69 kDa) and gBc (58 kDa) (Fig. 3C). gBa and gBb of C-terminally truncated variants revealed the expected decreased molecular masses compared to that of PrV WT gB or gB-N735S (Fig. 3C). Western blot analysis demonstrated the presence of both subunits gBb and gBc for all constructs, indicating that introduction of the N735S mutation did not impair gB processing (Fig. 3C).

Total cellular and surface expression of the gB mutants was analyzed by indirect immunofluorescence of transfected RK13 cells after or without permeabilization (Fig. 3D). While PrV WT gB and gB-N735S were barely detectable on the cell surface, PrV gB^ΔCTD1^, gB^ΔCTD2^, and gB^ΔCTD3^ were readily observed independent of the N735S mutation. The enhanced surface expression of the gB CTD truncation mutants correlates with previous reports (13) and can be attributed to the lack of two functional endocytosis motifs (^890^LL^891^ and ^905^YQRL^908^) in the gB CTD (Fig. 3A). Overall, introduction of the N735S mutation had no apparent effect on the subcellular localization of the respective gB variants (see Fig. S3) and does not impact gB processing.

10.1128/mBio.00557-21.3 FIG S3 Subcellular localization of PrV gB mutants with C-terminal truncations and N735S mutation. Representative images of the subcellular localization of the different gB variants in fixed and permeabilized RK13 cells 18 h posttransfection (Leica DMi6000 TZS SP5, Leica Microsystems, Wetzlar, Germany). PrV gB was detected using PrV gB-specific rabbit antiserum and Alexa Fluor 488-conjugated secondary antibodies (green). Nuclei were stained with Hoechst 33342 (blue). Bars, 10 nm. Download FIG S3, TIF file, 2.1 MB. Copyright © 2021 Vallbracht et al. 2021 Vallbracht et al. https://creativecommons.org/licenses/by/4.0/ This content is distributed under the terms of the Creative Commons Attribution 4.0 International license.

### Loss of two endocytosis motifs in gB^ΔCTD2^N735S is responsible for the hyperfusogenic phenotype but not for gH/gL independence.

**FIG 4:**
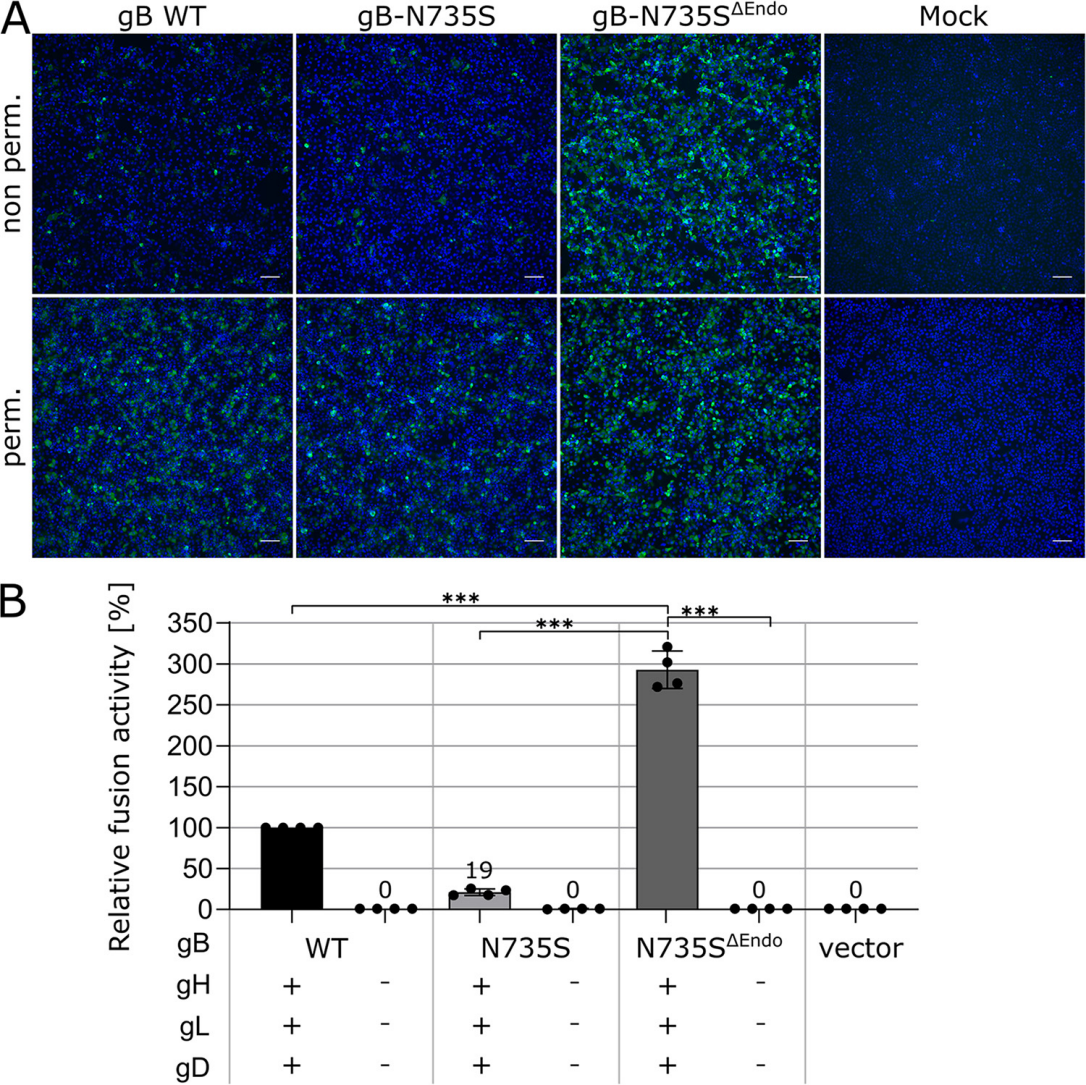
Hyperfusogenicity and gH/gL-independent fusion are unrelated features. (A) Total and surface expression of PrV gB WT, gB-N735S, and gB-N735S^ΔEndo^ were analyzed by indirect immunofluorescence 18 h posttransfection of RK13 cells using a Leica DMi8 microscope (Leica Microsystems, Wetzlar, Germany). PrV gB was detected using a specific rabbit antiserum and Alexa Fluor 488-conjugated secondary antibodies (green). Nuclei were counterstained with Hoechst 33342 (blue). Bars, 100 nm. (B) Fusion activity of the PrV gB variants in presence or absence of gH/gL and gD. Relative fusion activities compared to that of gB WT were determined 18 h posttransfection of RK13 cells. Mean relative values and standard deviations are shown (*n* = 4). Mean values below 50% are indicated. Two-tailed Welch’s *t* test; ***, *P* < 0.001.

To clarify whether the hyperfusogenic phenotype and/or gH/gL independence of gB^ΔCTD2^N735S is due to enhanced surface expression, we inactivated both endocytosis motifs (^890^LL^891^ and ^905^YQRL^908^) (Fig. 3A) in the gB CTD of full-length gB-N735S (gB-N735S^ΔEndo^) by alanine substitutions (LL^890/891^AA and Y^905^A). Immunofluorescence analyses revealed significantly enhanced surface expression of gB-N735S^ΔEndo^ as seen for the C-terminally truncated gB variants, demonstrating the functionality of the motifs (Fig. 3A and 4A). Concomitant with the enhanced surface expression, fusion activity of gB-N735S^ΔEndo^ was significantly increased (∼300% of that for gB WT) (Fig. 4B). Nevertheless, despite the hyperfusogenic phenotype, gB-N735S^ΔEndo^ was not able to mediate gH/gL-independent membrane fusion. These data indicate that (i) the hyperfusogenic phenotype observed for gB^ΔCTD2^N735S is due to the lack of two functional endocytosis motifs but that (ii) hyperfusogenicity *per se* is not sufficient for gH/gL-independent fusion, requiring both the N735S mutation and a short or absent gB CTD.

### A common mechanism of fusion activation of different alphaherpesvirus gBs.

**FIG 5:**
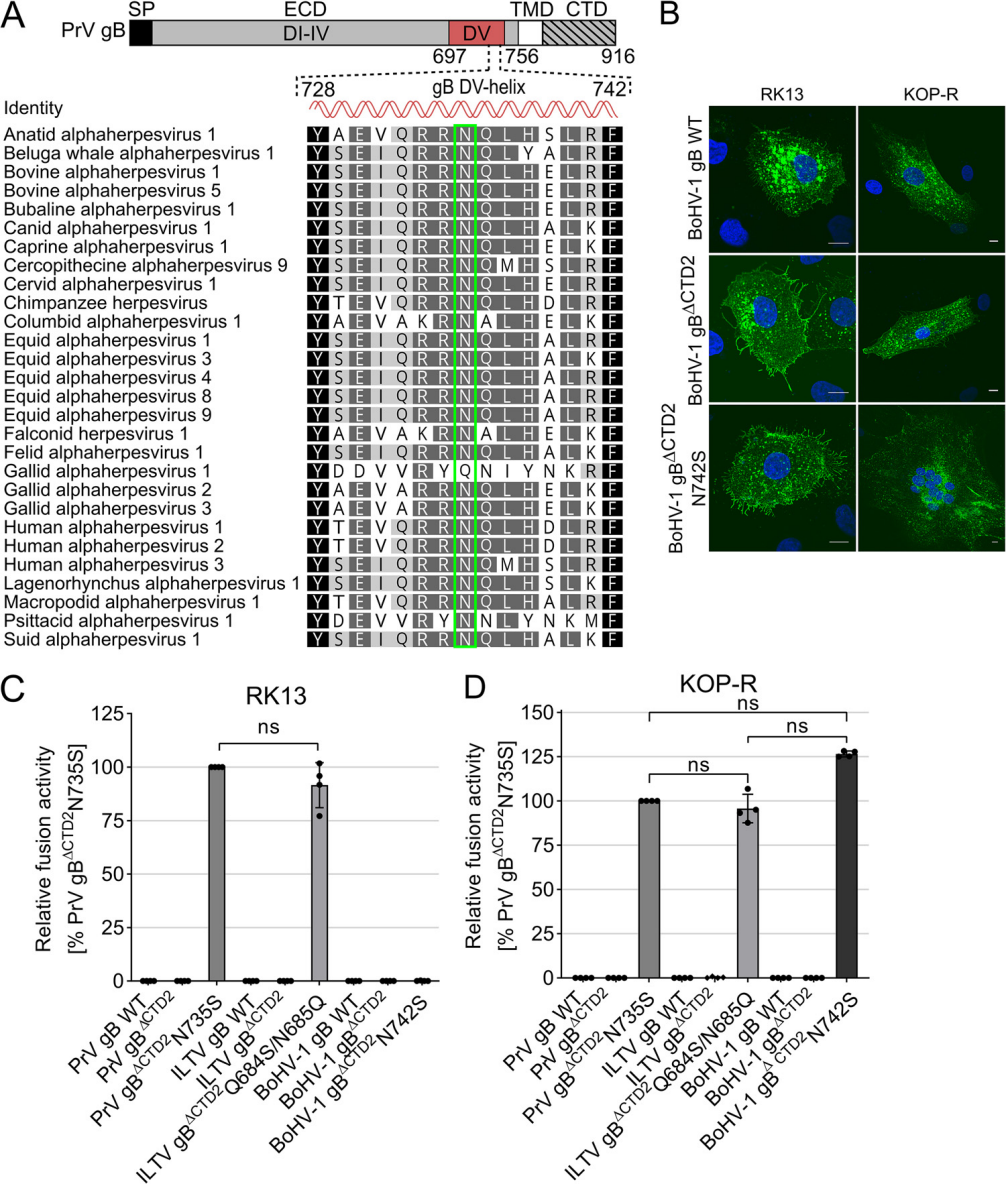
Asparagine in the gB DV helix is highly conserved, and mutation to serine leads to autonomous fusogenicity of different gB homologs. (A) Alignment of amino acid sequences of gBs from 28 different alphaherpesviruses was generated using the ClustalW plug-in of Geneious (Geneious Prime 2019 2.3). Only the residues forming the conserved regulatory helix in domain V of postfusion PrV gB (aa 697 to 756) and corresponding sequences of the other alphaherpesvirus gBs are shown and colored according to their similarity. Black, 100%; dark gray, <100% to 80%; light gray, <80% to 60%; white, <60%. N735 of PrV gB (*Suid alphaherpesvirus 1*) (bottom) and corresponding amino acids of the other gB homologs are boxed in green. Corresponding GenBank accession numbers top to bottom: YP_003084394.1, ASW27079.1, ALR87798.1, AAD46112.2, APO15888.1, AEK27122.1, AAD46114.2, NP_077446.1, AAD46115.2, BAE47051.1, YP_009352935.1, AII81366.1, YP_009054936.1, NP_045250.1, YP_006273012.1, YP_002333514.1, YP_009046525.1, ANG65542.1, AFD36568.1, YP_001033956.1, NP_066859.1, AAF04615.1, ADG45133.1, AAP32845.1, BAM99305.1, AAD11960.1, NP_944400.1, AEM64049.1. (B) Indirect immunofluorescence analysis of BoHV-1 WT gB and mutant gB 18 h after transfection of RK13 (left) or KOP-R cells (right). BoHV-1 gB was detected using the monoclonal antibody 42/18/7 and Alexa Fluor 488-conjugated secondary antibodies (green). Nuclei were stained with Hoechst 33342 (blue). Representative images of fixed and permeabilized RK13 (left) and KOP-R cells (right) are shown (Leica DMi6000 TZS SP5, Leica Microsystems, Wetzlar, Germany). Bars, 10 nm. (C and D) Fusion activity of the PrV, ILTV, and BoHV-1 gB variants. Relative fusion activity was determined 18 h posttransfection of RK13 (C) or KOP-R cells (D) with the corresponding expression plasmids. Mean relative values and standard deviations are shown (*n* = 4). Two-tailed Welch’s *t* test; ns, not significant.

Sequence analyses revealed that N735 is highly conserved across alphaherpesvirus gBs (Fig. 5A). The high sequence conservation prompted us to investigate whether mutation of this site in addition to gB CTD truncation could transform other gB homologs into autonomous fusion proteins. To this end, we focused on the closely related *Varicellovirus* BoHV-1 and the distant avian ILTV of the *Iltovirus* genus. Sequence analyses and secondary structure prediction using JPred4 revealed that, similar to PrV gB, BoHV-1 and ILTV gB contain two extended putative alpha-helices in the CTD (Fig. S1). Therefore, BoHV-1 and ILTV gB were truncated at sites corresponding to K857 (ΔCTD2) of PrV gB, resulting in removal of both predicted alpha-helices (Fig. S1). N742 of BoHV-1 gB corresponds to N735 of PrV gB and was replaced by serine. Interestingly, in the more distant ILTV gB, the highly conserved NQ dipeptide motif is inverted to QN (positions 684/685) (Fig. 5A) and was replaced by SQ to match the sequence of PrV gB N735S (ILTV gB^ΔCTD2^Q684S/N685Q). The resulting gB mutants were tested for their ability to mediate membrane fusion on various immortalized and primary cells (Fig. 5C and D; see also Fig. S4).

10.1128/mBio.00557-21.4 FIG S4 PrV and ILTV gB mutants mediate autonomous fusion on various cells. Fusion activities of PrV, ILTV, and BoHV-1 WT and mutant gBs were determined 24 h posttransfection of embryonic porcine kidney epithelial inoculated line (SPEV), human embryonic kidney 293T (HEK293T), human neuroblastoma (SK-N-SH), human melanoma (MeWo), primary chicken and turkey embryo kidney (CEK and TEK) cells or African green monkey kidney (Vero) cells with the corresponding gB expression plasmids. Assays with empty vector served as a negative control. Mean absolute values and standard deviations from three independent experiments are shown (*n* = 3). Download FIG S4, TIF file, 1.1 MB. Copyright © 2021 Vallbracht et al. 2021 Vallbracht et al. https://creativecommons.org/licenses/by/4.0/ This content is distributed under the terms of the Creative Commons Attribution 4.0 International license.

Neither BoHV-1 gB^ΔCTD2^ nor ILTV gB^ΔCTD2^ was fusion active, again demonstrating the importance of the CTD for membrane fusion and highlighting that the C-terminal truncation *per se* cannot compensate for gH/gL function during fusion. Remarkably, similar to PrV gB^ΔCTD2^N735S, ILTV gB^ΔCTD2^Q684S/N685Q revealed an autonomous, hyperfusogenic phenotype on RK13, bovine esophagus (KOP-R) (Fig. 5C and D), and several other cells, including primary turkey and chicken embryo kidney cells (TEK and CEK cells, respectively) (Fig. S4). In contrast, autonomous fusion by BoHV-1 gB^ΔCTD2^N742S was restricted to cells of bovine origin, including KOP-R cells (Fig. 5C and D) and Madin-Darby bovine kidney (MDBK) epithelial cells (Fig. S4), suggesting that the BoHV-1 gB^ΔCTD2^N742S requires a signal specific to bovine cells. To exclude that lack of fusion activity of BoHV-1 gB^ΔCTD2^N742S on nonbovine cells was due to impaired protein expression, immunofluorescence analyses of transfected RK13 and KOP-R cells were performed (Fig. 5B). Expression of BoHV-1 WT gB and the C-terminally truncated BoHV-1 gB variants was detected on both RK13 and KOP-R cell lines, demonstrating that lack of fusion activity was not due to impaired protein expression (Fig. 5B). Overall, these results demonstrate that mutation to serine of the conserved asparagine residue in the putative DV helix of C-terminally truncated BoHV-1 and ILTV gB allows for gH/gL-independent fusion, indicating functional conservation of the mutated region and strongly suggests a common central role of the regulatory helix for the fusion mechanism of alphaherpesvirus gB.

### Fusion regulation of HSV-1 gB differs from that of other alphaherpesvirus gBs.

**FIG 6:**
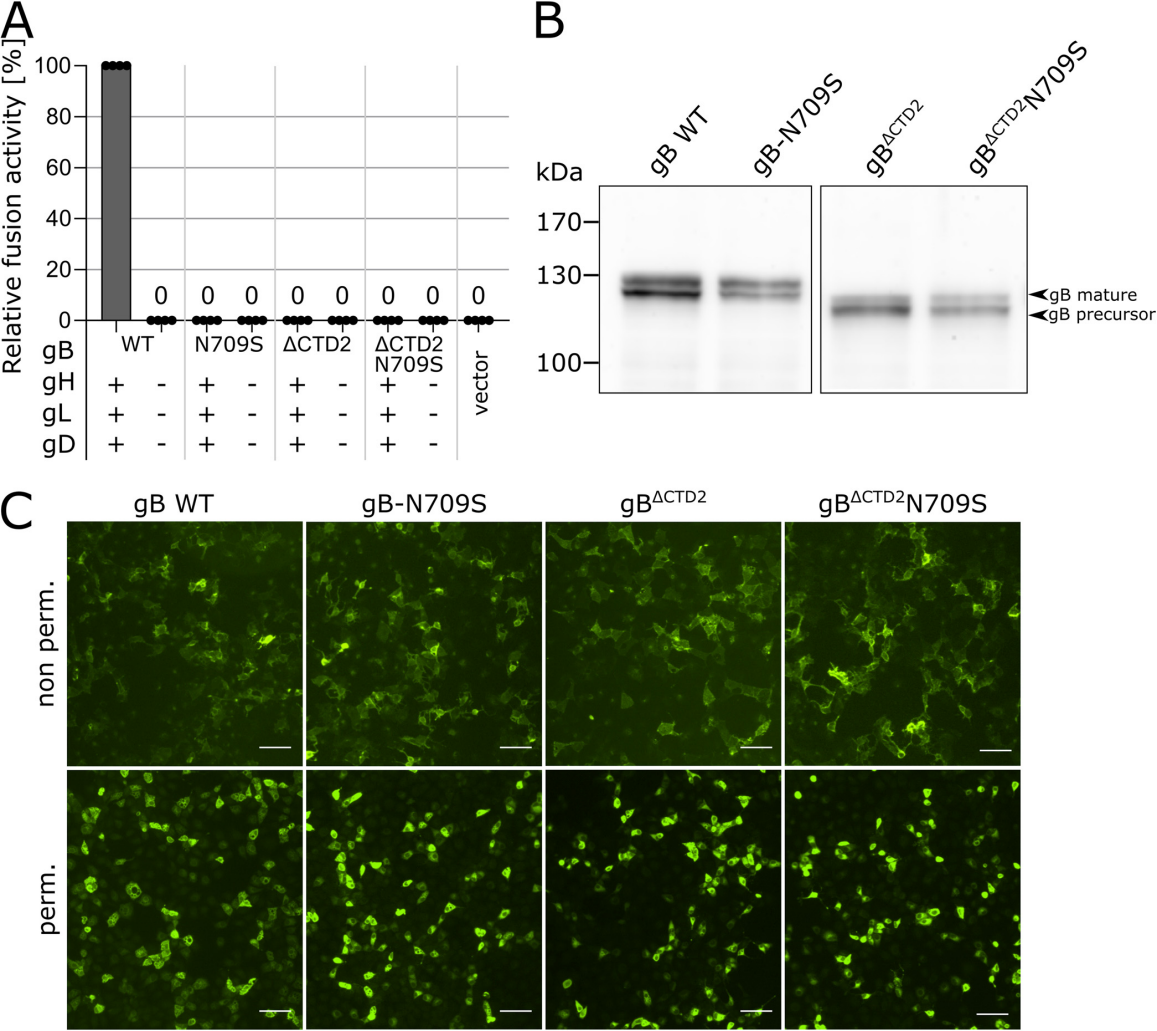
The conserved asparagine residue in DV is crucial for HSV-1 gB fusogenicity, but substitution to serine does not confer gH/gL independence. (A) Fusion activity of HSV-1 WT gB, gB-N709S, and gB^ΔCTD2^N709S in presence or absence of HSV-1 gH/gL and gD. Relative fusion activities of the HSV-1 gB variants was determined 24 h posttransfection with the corresponding expression plasmids. Mean relative values and standard deviations are shown (*n* = 4). Zero (0) indicates absence of fusion activity. (B) Western blot analyses of HSV-1 gB variants 24 h after transfection of RK13 cells. HSV-1 gB was detected using a specific rabbit antiserum and peroxidase-conjugated secondary antibody. Signals of mature gB (upper band) or precursor gB (lower band) are marked, and the molecular masses of marker proteins are indicated. (C) Total and surface expression of HSV-1 WT gB and mutant gB were analyzed by indirect immunofluorescence 24 h after transfection of RK13 (Leica DMi6000 TZS SP5, Leica Microsystems, Wetzlar, Germany). HSV-1 gB was detected using HSV-1 gB-specific rabbit antiserum and Alexa Fluor 488-conjugated secondary antibodies (green). Bars, 100 nm.

Since asparagine in the DV helix is also conserved in members of the *Simplexvirus* genus (Fig. 5A), we tested whether fusogenicity of HSV-1 gB might be regulated by a similar mechanism. To this end, we truncated HSV-1 gB at residue K839 corresponding to K857 (ΔCTD2) in PrV gB (Fig. S1) and replaced N709, corresponding to N735 in PrV gB with serine (HSV-1 gB^ΔCTD2^N709S). In addition, the N709S mutation was also introduced into full-length HSV-1 gB (HSV-1 gB-N709S). These mutants were tested for their ability to mediate cell-cell fusion in the presence or absence of gH/gL (Fig. 6A). Comparable to PrV gB^ΔCTD2^, truncation of HSV-1 gB at K839 completely abolished fusion activity independent of the presence or absence of gH/gL and gD (Fig. 6A). However, in contrast to PrV, BoHV-1, and ILTV gB mutants (Fig. 5C and D), insertion of N709S was not able to rescue fusogenicity of HSV-1 gB^ΔCTD2^, and none of the HSV-1 gB variants revealed gH/gL-independent fusion activity (Fig. 6A). Nevertheless, like in PrV gB-N735S (Fig. 3B), introduction of N709S into full-length HSV-1 gB led to significantly decreased fusion levels (Fig. 6A), demonstrating that conserved asparagine in the DV helix indeed plays an important role for HSV-1 gB function. Lack of fusogenicity of these mutants was not due to impaired protein expression, as evident from Western blot and immunofluorescence analyses showing that all mutants are properly processed (Fig. 6B) and expressed on the cell surface (Fig. 6C). Overall, these results suggest that, despite the high sequence conservation, fusion by HSV-1 gB might be regulated by a different mechanism, highlighting intriguing differences between the here-investigated animal alphaherpesviruses (PrV, BoHV-1, and ILTV) and human HSV-1.

## DISCUSSION

In this study, we investigated the activation control of the herpesvirus fusogen gB. Our data identify a conserved “regulatory” helix in gB DV that may act as a central “switch” for the pre-to-postfusion conformational change in alphaherpesviruses. Our results support a model in which the DV helix is directly involved in the structural signal transduction between the gB endo- and ectodomains, enabling the functional allosteric coupling between these two domains for fusion regulation.

**FIG 7:**
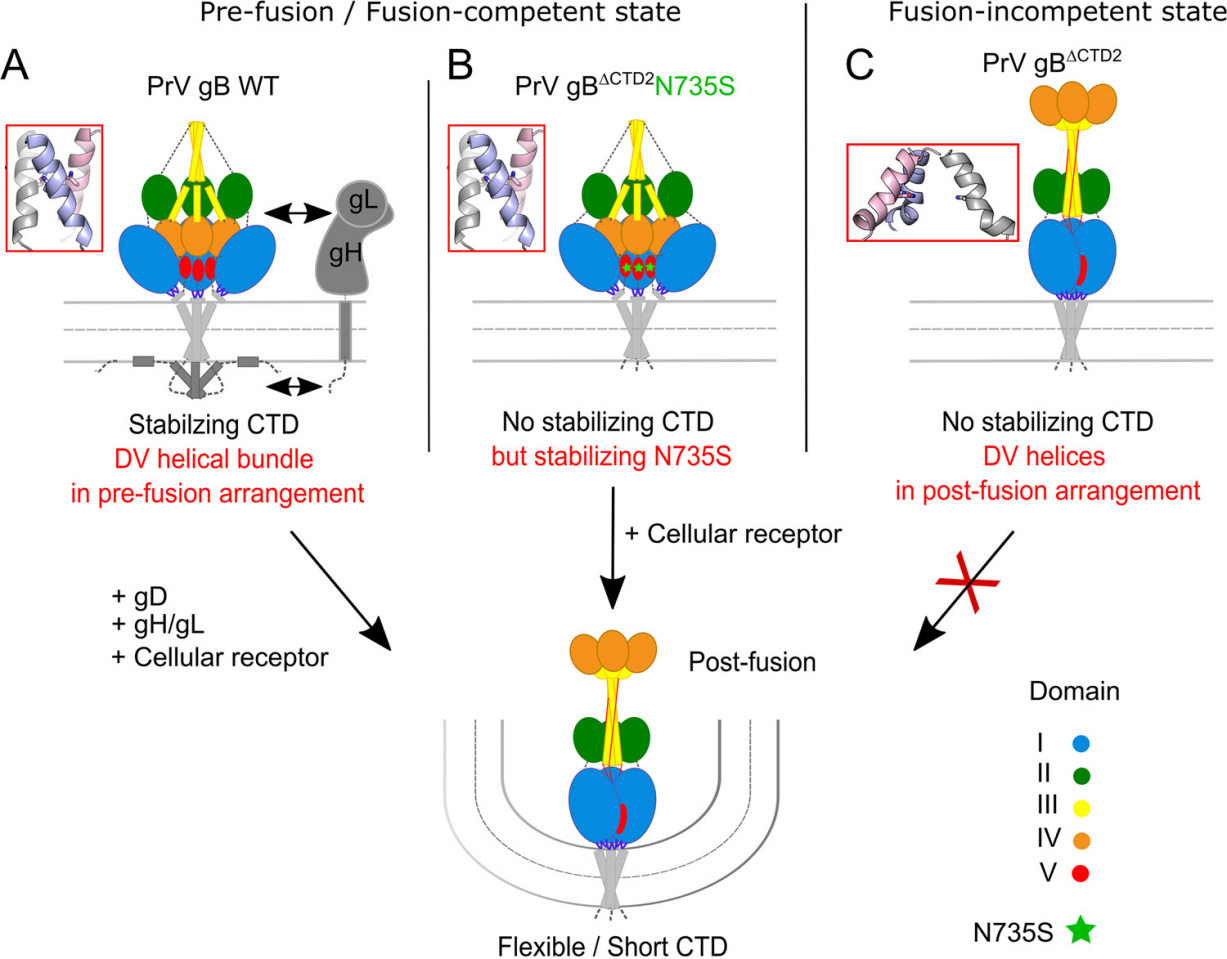
Model of the gB endo- and ectodomain cross talk involving the regulatory DV helical bundle as a switch for the pre-to-postfusion structural transition. (A) Stable membrane interaction by the gB cytoplasmic domain (CTD) and presence of gH/gL are required to maintain PrV WT gB in a metastable prefusion conformation and the regulatory DV helical bundle is in a prefusion state. Fusogenic refolding of WT gB requires release of the gB CTD clamp and rearrangement of the DV helical bundle via activated gH/gL and gD. (B) N735S mutation provides stability of the DV helical bundle thereby allowing PrV gB^ΔCTD2^N735S to adopt a fusion-competent state. The respective fusion-active form is decoupled from regulation by the CTD and ectodomain cross talk and does not require gH/gL for activation. gB conformational change into the stable postfusion state is triggered by interaction with cellular receptor(s). N735S mutation is marked by green asterisks. (C) PrV gB^ΔCTD2^ lacking a stabilizing CTD and N735S mutation is unable to adopt a stable fusion-competent conformation leading to a defect in membrane fusion.

Using *in vitro* evolution of an entry-deficient PrV mutant expressing a C-terminally truncated gB (PrV-gB^ΔCTD2^), we isolated an infectious revertant (PrV-gB^ΔCTD2^Pass) expressing a gB variant that mediates highly efficient autonomous *in vitro* cell fusion as well as gH/gL-independent viral entry due to a single amino acid substitution in the ectodomain (N735S) (Fig. 2). Whereas the C-terminally truncated gB variants of different alphaherpesvirus gB homologs (gB^ΔCTD2^) were inactive in fusion despite proper processing and efficient surface expression (Fig. 3B to D), engineering this substitution (PrV, N735S; BoHV-1, N742S; ILTV, Q684S/N685Q) into the gB ectodomain compensated for the lack of the gB CTD and allowed for gH/gL independence during membrane fusion (Fig. 2 and 5C and D). The fact that a single point mutation in the ectodomain can compensate for absence of part or the complete cytoplasmic domain and gH/gL strongly supports the notion of finely tuned functional cross talk between these domains for fusion regulation, as was proposed previously (20, 37). Considering the recently proposed role of the CTD in maintaining the prefusion conformation (12, 14, 23, 24, 38), it is conceivable that the C-terminally truncated gB^ΔCTD2^ and gB^ΔCTD3^ tested here, similar to soluble gB ectodomains (4, 22), adopt the postfusion conformation before reaching the cell surface, resulting in the observed null-fusion phenotype (Fig. 3B and 7). The N735S mutation, in turn, might have been selected because it maintains the ectodomain in a fusion-competent state in the absence of the CTD (Fig. 7). In the presence of the entire or a large part of the gB CTD, as in WT gB and gB^ΔCTD1^, the N735S mutation would stabilize the prefusion form to an extent where gH/gL would again be necessary for its activation, explaining the reduced and gH/gL-dependent fusion activity of gB-N735S and gB^ΔCTD1^N735S (Fig. 3B). Transcomplementation studies of gB/gH-deficient PrV mutants, whose infectivity was rescued by gB^ΔCTD2^N735S but not by WT gB (Fig. 2B), support the hypothesis that the N735S mutation acts by stabilizing PrV gB in a fusion-competent state that can be triggered in the absence of the gH/gL complex. Importantly, these data suggest that the DV helix is directly involved in the structural signal transduction between the endo- and ectodomains in WT gB and thereby enables their functional cross talk for fusion activation. In gB^ΔCTD2^N735S, this cross talk is overridden, leading to gH/gL-independent fusion.

Based on our findings, we suggest that (i) the gB CTD and/or the N735S mutation stabilizes the gB ectodomain in a fusion-active conformation, (ii) the gB CTD renders the molecule dependent on gH/gL for fusion, indicating that (iii) the gB CTD is not required for gB fusogenicity *per se* but provides a way of controlled triggering of the fusogenic conformational change involving the DV helix as a communicator, as proposed below.

**FIG 8:**
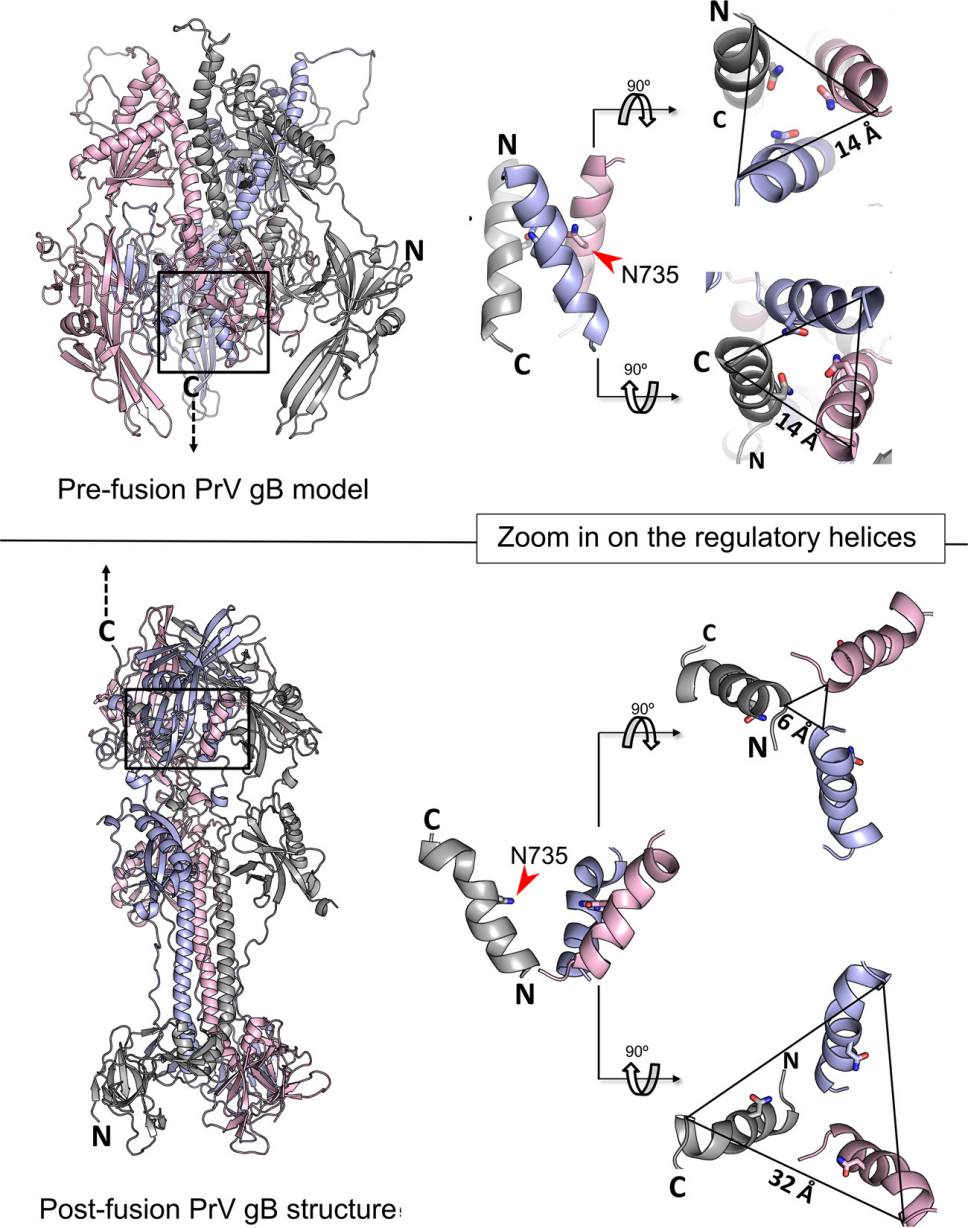
Conformational changes within the DV helical bundle during the pre-to-postfusion transition. (Top left) PrV gB ectodomain prefusion model was generated using Modeller (63) with the HSV-1 gB prefusion structure as a template (25). (Bottom left) Ribbon diagram of the PrV gB postfusion ectodomain (6ESC). Position of the regulatory helical bundle in domain V (DV) of pre- and postfusion gB is indicated by black boxes. Zoom in on the regulatory DV helical bundle in prefusion (top right) and postfusion (bottom right) gB. Side chain of N735 is shown and the distances of the N and C termini of the individual DV helices of the pre- and postfusion trimers are indicated. Structure images were generated using PyMOL (64).

N735S maps to the interface of the PrV gB postfusion trimer and is part of a short conserved alpha-helix (Fig. 1D and 5A) (4). So far, HSV-1 gB is the only homolog for which there is information on the prefusion conformation at a resolution that allows placement of DV (25). The high sequence conservation between PrV and HSV-1 gB (3) warranted the use of the HSV-1 gB prefusion structure as a template for the generation of a theoretical model of the PrV gB ectodomain in prefusion form (Fig. 8, top). The model shows that, as in HSV-1 gB, PrV gB DVs form a bundle at the interior of the trimer-interface, close to the viral membrane. The side chains of N735 are predicted to interact at the trimer interface (Fig. 8, top). We propose that rearrangement of this regulatory DV helix is key to the fusogenic refolding of gB (Fig. 7 and 8). The DV helical bundle would need to be tight enough to prevent the spontaneous conformational change but labile enough to allow it when the CTD is activated. DV continues into the membrane-proximal region (MPR), transmembrane anchor (TMD), and the CTD (not modeled), and we hypothesize that the effects of the mutations in the regulatory helices impacting their arrangement/packing could be propagated via the MPR and the TMD to the CTD, effectively decoupling the CTD and ectodomain cross talk. The fact that the hyperfusogenic gH/gL-independent phenotype was able to be transferred to gB of the closely related *Varicellovirus* BoHV-1 and gB of the more distant *Iltovirus* ILTV (Fig. 5C and D) suggests that this control mechanism may be common to alphaherpesvirus gBs. Although our attempts to generate a gH/gL-independently acting HSV-1 gB mutant failed, our data highlight a crucial role of the DV helix and, in particular, the conserved asparagine residue for HSV-1 gB-mediated fusion. Thus, while introduction of N709S mutation into HSV-1 gB^ΔCTD2^ did not rescue the lack of the CTD, N709S completely abolished fusion in the context of full-length HSV-1 gB (Fig. 6A) without affecting protein expression (Fig. 6B and C).

Notably, gB variants from two gammaherpesviruses, Epstein-Barr virus (EBV) and Kaposi's sarcoma-associated herpesvirus, with C-terminal truncations or point mutations induce very low levels of *in vitro* cell-cell fusion independent of gH/gL due to increased surface expression or elevated temperatures (39–42), highlighting potential differences in the fusion control mechanisms between gB of the two subfamilies. While we showed that enhanced surface expression due to inactivation of two functional endocytosis motifs contributes to the high fusion levels of gB^ΔCTD2^N735S and gB^ΔCTD3^N735S (Fig. 2A and 3B), enhanced surface expression or C-terminal truncation *per se* were not sufficient for gH/gL-independent fusion (Fig. 3B, 4, and 5C and D). Thus, the alphaherpesvirus gB mutants investigated in this study clearly differ from the previously reported gammaherpesvirus gB mutants, in that elevated surface levels or CTD truncations of alphaherpesvirus gB are not sufficient for gH/gL-independent fusion.

We found that membrane fusion by BoHV-1 gB^ΔCTD2^N742S requires activation by a trigger specific to bovine cells (Fig. 5C and D) whose identification will await further studies. Thus, while species-specific receptor engagement may serve as a fusion trigger for BoHV-1 gB, it is conceivable that PrV and ILTV gB utilize a more ubiquitous receptor(s) or signals, which, at least for PrV, reflect its broad host tropism (43). Since specific bovine cell lines are a prerequisite for autonomous fusion by BoHV-1, it cannot be excluded that HSV-1 gB requires specific cells for gH/gL-independent fusion which have not yet been identified. In the current model for alphaherpesvirus entry, gD acts as the principal receptor-binding protein that determines cell tropism and triggers viral entry (44, 45). In addition to gD, receptors for alphaherpesvirus gH/gL have been described, and a direct role in activation of the fusion cascade was proposed (46, 47). The results presented here indicate that alphaherpesvirus gB may not only catalyze fusion but itself may play a more direct role in triggering fusion via receptor engagement, highlighting the potential of gB as tropism determinant for alphaherpesvirus entry. Soluble forms of BoHV-1 and HSV-1 gB have been shown to inhibit entry, demonstrating a relevant role for gB receptor engagement during cell invasion (48, 49). Receptors for alphaherpesvirus gB have been identified (50–54), but their role in triggering conformational changes in gB is unknown. For BoHV-1 gB, binding to an as-yet-unknown non-heparan sulfate receptor has been demonstrated to be critical for entry and dependent on the presence of the CTD (48, 55). While it needs to be investigated whether receptor binding by gB plays a role in triggering its conformational change for viral entry in the presence of gH/gL, receptor engagement by gB could play an additional role, e.g., in entry efficiency or signal transduction to promote viral dissemination or persistence. It is interesting to speculate that a possible progenitor virus might have been able to enter cells using gB as a stand-alone fusion protein. The acquisition or evolution of gH/gL to bind additional receptors and to trigger gB-mediated fusion may then have allowed herpesviruses to infect new hosts, contributing to the success of this large family of complex DNA viruses. Overall, the findings are also consistent with the observation that the class III structural homologs of gB triggered by acidic pH lack a structured endodomain, as additional control is not required for entry of those viruses (see Fig. S1 in the supplemental material).

For HCMV, it was hypothesized that gH/gL might stabilize gB in its prefusion state, making structural studies on prefusion gB challenging (24). It will be interesting to investigate whether further stabilization of the DV helical bundle could generate more stable prefusion gB variants, which would be valuable as a target for vaccine or drug design. The high structural, sequence, and functional conservation of the DV helix suggests that it might be potentially vulnerable to pharmacological interventions. In addition, the autonomously acting gB mutants described here offer a novel platform allowing for direct investigations of the central fusogenic conformational change of gB, now uncoupled from the function of the other entry glycoproteins. This also opens up a new window of opportunity to identify cellular gB receptors that can trigger gB conformational change during fusion and viral entry.

Whether gB has the intrinsic ability of driving fusion on its own has been a long-standing question, and our results reveal that it is the allosteric coupling between the gB endo- and ectodomain that explains the control exerted by gH/gL and gD. Overall, our study expands the understanding of the elements controlling the conformational change of gB resulting in fusion during herpesvirus entry and resolves a fundamental conundrum in herpesvirus biology. Thus, it is of fundamental virological interest and has a considerable potential to guide the development of novel prevention and treatment options against herpesvirus infections.

## MATERIALS AND METHODS

### Cells.

Cell lines were obtained from the Cell Culture Collection in Veterinary Medicine (CCLV) of the FLI. Rabbit kidney (RK13; CCLV-RIE 0109), Madin-Darby bovine kidney (MDBK; CCLV-RIE 0261), bovine esophagus (KOP-R; CCLV-RIE 0244), African green monkey kidney (Vero; CCLV-RIE 0228), embryonic porcine kidney (SPEV; CCLV-RIE 0008), human melanoma (MeWo), and human embryonic kidney 293T (HEK293T; CCLV-RIE 1018) cells were grown in minimum essential medium (MEM) supplemented with 10% fetal calf serum (FCS) at 37°C in a saturated atmosphere containing 5% CO_2_. Primary chicken and turkey embryo kidney (CEK and TEK) cells were prepared according to standard procedures (56). The human neuroblastoma cell line SK-N-SH (ATCC HTB-11) was grown in Eagle’s minimum essential medium supplemented with 10% FCS at 37°C in a saturated atmosphere containing 5% CO_2_. RK13 cell lines stably expressing gB (RK13-gB) (13), gB and gD (RK13-gB/gD) (35), or gB and gH/gL (RK13-gB/gH/gL) (35) of Pseudorabies virus (PrV; *Suid alphaherpesvirus 1*) strain Kaplan (Ka) (57) were grown in MEM supplemented with 10% FCS and 500 μg/ml G418 (Invitrogen, Karlsruhe, Germany).

For RK13 cell lines expressing gB^ΔCTD2^N735S or PrV WT gB under the authentic promoter, the gB open reading frame (ORF) including the core-promoter region was amplified from genomic PrV gB^ΔCTD2^Pass and PrV Ka DNA using primer pairs PrV-gBPro_for/gBC-37_rev and PrV-gBPro_for/gB_Ass_rev (see Table S1 in the supplemental material). PCR products were cloned into pcDNA3 (Invitrogen). Plasmids were used to transfect RK13 cells, and positive cell clones were selected with G418 (Invitrogen) and identified by immunofluorescence microscopy after infection with PrV-ΔgB.

10.1128/mBio.00557-21.5 TABLE S1 Primers used in this study. Nonmatching nucleotides are in italics, restriction sites used for cloning are underlined, and mutations introduced by site-directed mutagenesis are in bold. Download Table S1, DOCX file, 0.01 MB. Copyright © 2021 Vallbracht et al. 2021 Vallbracht et al. https://creativecommons.org/licenses/by/4.0/ This content is distributed under the terms of the Creative Commons Attribution 4.0 International license.

### Viruses.

Viruses used in this study were derived from PrV Ka (57). PrV-gB^ΔCTD2^ used for the passaging experiments was propagated on PrV gB-expressing cells (13). PrV deletion mutants simultaneously lacking gB and gH (PrV-ΔgB/H) (35), gB and gD (PrV-ΔgB/D) (35), or only gB (PrV-ΔgB) (13) were propagated on complementing RK13 PrV-gB/gH/gL (35), RK13 PrV-gB/gD (35), or RK13 PrV-gB (13) cell lines, respectively.

### Passaging of PrV-gB^ΔCTD2^ and isolation of PrV-gB^ΔCTD2^Pass.

PrV-gB^ΔCTD2^ was passaged as described previously (26). Briefly, RK13 cells were infected with phenotypically gB-complemented PrV-gB^ΔCTD2^ at a multiplicity of infection (MOI) of 0.01 and trypsinized as soon as the monolayer reached 100% confluence. Infected cells were repeatedly coseeded with noninfected RK13 cells until infectivity was stably detectable in the supernatant. Supernatants of all passages were cleared from cell debris by low-speed centrifugation and titrated on RK13 cells. Precleared supernatant of the 19th passage was used to infect RK13 cells. Infected cells from individual plaques were aspirated with a pipet tip and used for infection of RK13 cells grown in 75-cm^2^ flasks for further isolation and propagation of the revertant viruses. After a complete cytopathic effect (CPE) was observed, cells were lysed by freeze-thawing and centrifuged for 10 min at 4,000 rpm. Viral DNA was isolated from the cell pellet as previously described (58), and supernatants were stored at −80°C. Genomic DNA was analyzed by restriction analysis (results not shown) and used for amplification of the gD, gH, gL, and gB ORFs. The PCR products were subsequently sequenced.

### Expression plasmids.

Expression plasmids for PrV glycoproteins have been generated as described previously using PrV Ka DNA as the template (13). PrV gB residues are numbered according to GenBank accession number AEM64049.1. N735S mutation was introduced by site-directed mutagenesis (Agilent Technologies; QuikChange II site-directed mutagenesis kit) using primers PrVgB-N735S-F and PrVgB-N735S-R (Table S1). The same method and primer pairs, PrV-gB-LL890/891AA-F with PrV-gB-LL890/891AA-R and PrV-gBY905A-F with PrV-gBY905A-R, were used to generate the PrV gB mutants with inactivation of the C-terminal endocytosis motifs (Table S1). gB of avian infectious laryngotracheitis virus (ILTV; *Gallid alphaherpesvirus 1*) was cloned from genomic DNA of virulent laboratory strain ILTV A489 (58), which is closely related to the fully sequenced USDA reference strain (GenBank accession JN542534). The coding region was amplified using primers IgB-F1 and IgB-R1 and cloned into pcDNA3I (59) (Table S1). ILTV gB mutants were generated by site-directed mutagenesis using primer pairs IgBtrunc-F/IgBtrunc-R and/or IgBSQ-F/IgBSQ-R (Table S1).

The ORFs encoding gB (UL27), gH (UL22), gL (UL1), and gD (US6) of *Bovine herpesvirus 1* (BoHV-1) were cloned from genomic DNA of BoHV-1 strain Schönböken (kindly provided by P. König, FLI, Greifswald-Insel Riems, Germany), which is closely related to the fully sequenced BoHV-1 reference strain Cooper (GenBank accession number KU198480). The coding regions were amplified using primer pairs BoHV-1-gB_for/BoHV-1-gB_rev for gB, BHV-gH_for/BHV-gH_rev for gH, BHV-gL_for/BHV-gL_rev for gL, and BHV-gD_for/BHV-gD_rev for gD (Table S1). Purified PCR products were cloned into pcDNA3 (Invitrogen) via restriction sites introduced by the primers. The BoHV-1 gB residues are numbered according to GenBank accession number ALR87798.1. C-terminally truncated BoHV-1 gB^ΔCTD2^ was generated by PCR using the expression plasmid for BoHV-1 WT gB as the template and primer pair BoHV-1-gB_for/BoHV-1-gB^ΔCTD2^EcoRI (Table S1). The purified PCR product was cloned into pcDNA3 via BamHI and EcoRI restriction sites that were introduced by the primers (Table S1). The N742S mutation was introduced into truncated BoHV-1 gB^ΔCTD2^ by site-directed mutagenesis (QuikChange II kit; Agilent) using primer pair BoHV-1-gBN742S_F/BoHV-1-gBN742S_R (Table S1).

Plasmids for HSV-1 gB, gL, and gD have been described previously. N709S was introduced via QuikChange mutagenesis, and the C-terminally truncated variant was generated via PCR using primer pair HSV-1-gB-F and HSV-1-gBK839*. The purified PCR product was cloned into pcDNA3 via BamHI and EcoRI restriction sites that were introduced by the primers (Table S1). Correct sequences of the inserts of all generated plasmids were verified by DNA sequencing using T7, SP6 primers as well as primers 130 and 134 (PrV gB), BoHV-1-gB_seq_T277 (BoHV-1 gB), HSV-1-gBR481, and HSV-1-gBA317 (HSV-1 gB) (Table S1).

### Sequence analyses.

Sequencing was performed using the BigDye Terminator v1.1 cycle sequencing kit and a 3130 Genetic Analyzer (Applied Biosystems). Results were evaluated using Geneious Prime software (version 2019 2.3).

### Plaque assays.

Approximately 8 × 10^5^ RK13 cells were seeded into 6-well plates and incubated at 37°C. On the following day, cells were infected with approximately 150 PFU/ml of PrV mutants or WT PrV for 1 h on ice to synchronize the infection. After 2 h at 37°C, the inoculum was replaced with semisolid medium containing 6 g/liter methylcellulose, and infected cells were incubated for 48 h at 37°C. Cells were subsequently fixed with 5% formaldehyde and stained with 1% crystal violet in 50% ethanol. Areas of 30 plaques per virus were measured using an Eclipse Ti-S fluorescence microscope and the NIS-Elements imaging software (Nikon, version 4). Percentages of WT PrV plaque sizes were calculated from three independent assays, and mean values and standard deviations were determined.

### Transcomplementation assay.

Approximately 2 × 10^5^ RK13-gBWT or RK13-gB^ΔCTD2^N735S cells were seeded into 24-well plates. After 24 h at 37°C, they were infected with phenotypically complemented PrV-ΔgB, PrV-ΔgB/H, or PrV-ΔgB/D at an MOI of 3 for 1 h on ice and subsequently for 2 h at 37°C. Thereafter, the inoculum was removed, and nonpenetrated virus was inactivated by low-pH treatment for 2 min. After three washing steps with phosphate-buffered saline, cells were overlaid with 1 ml fresh MEM supplemented with 5% FCS per well. After 24 h at 37°C, the cells were harvested, lysed by freeze-thawing (−80°C and 37°C), and centrifuged for 10 min at 4,000 rpm. Cell pellets were used for Western blot analyses, and virus supernatants were titrated on RK13-gBWT cells. Mean values from four independent experiments and corresponding standard deviations were determined.

### *In vitro* fusion assays.

The fusogenic properties of the different glycoproteins were assessed using a transient-transfection-based cell-fusion assay as described recently (34). Briefly, approximately 2 × 10^5^ cells/well were seeded into 24-well cell culture plates and transfected with 200 ng each of glycoprotein expression plasmids in different combinations, and a plasmid encoding enhanced green fluorescent protein (pEGFP-N1; Clontech) as a marker in 50 μl Opti-MEM using 1 μl Lipofectamine 2000 (Thermo Fisher Scientific). The transfection mixture was incubated for 20 min at room temperature and subsequently added to the cells. After 3 h at 37°C cells, were washed with phosphate-buffered saline (PBS), overlaid with fresh MEM supplemented with 2% FCS, and incubated for another 18 h at 37°C. Cells were fixed with 3% paraformaldehyde (PFA), and syncytium formation was assessed using an Eclipse Ti-S fluorescence microscope and the NIS-Elements imaging software (Nikon version 4). Cells with three or more nuclei were counted as syncytia. The total fusion activity was determined by multiplication of the mean syncytium area by the number of syncytia within 10 fields of view (5.5 mm^2^ each). Four independent assays were conducted, and mean values and standard deviations were determined.

### Indirect immunofluorescence analysis.

For indirect immunofluorescence analyses, RK13 cells were transfected (Lipofectamine 2000; Thermo Fisher Scientific) with the different gB expression plasmids and fixed 18 h posttransfection with 3% PFA in PBS for 20 min. Afterwards, cells were washed with PBS containing 50 mM NH_4_Cl for 30 min at room temperature. Optionally, PBS containing 0.1% Triton X-100 was used to permeabilize the cells for 10 min at room temperature before they were washed with PBS (3 × 5 min) and blocked with 0.25% milk in PBS for 30 min at room temperature. Subsequently, cells were incubated with primary antibodies for 1 h at 4°C. Bound antibody was detected using Alexa 488-conjugated secondary antibodies (Invitrogen) at a dilution of 1:1,000 in PBS. After each step, cells were washed with PBS three times for 5 min at room temperature. Representative images were taken with a Leica DMi8 fluorescence microscope (Leica Microsystems, Wetzlar, Germany) or a laser scanning confocal microscope (Leica DMi6000 TZS SP5, Leica Microsystems, Wetzlar, Germany).

### Western blot analyses.

Infected or transfected cells were harvested after 18 h and lysed, and protein samples were separated by discontinuous sodium dodecyl sulfate-polyacrylamide gel electrophoresis (SDS-PAGE) under reducing conditions and transferred to a nitrocellulose membrane. Membranes were incubated with appropriate antibodies. Peroxidase-conjugated secondary antibody (Jackson ImmunoResearch) was detected with Clarity Western ECL substrate (Bio-Rad) and recorded with a VersaDoc 4000 MP imager (Bio-Rad) using the Quantity One software (version 4.6.9).

### Antibodies, antisera, and staining solutions.

PrV gB was detected using a PrV gB-specific rabbit antiserum (60) at a dilution of 1:50,000 for Western blot analyses and 1:1,000 for immunofluorescence analyses. BoHV-1 gB was detected using BoHV-1 gB-specific monoclonal antibody (MAb) 42/18/7 (61) at a dilution of 1:200. Nuclei of fixed cells were stained with Hoechst (33342 staining dye solution; Abcam) at a dilution of 1:20,000 in PBS for 10 min at room temperature. In Western blot analyses, PrV gH was detected using a monospecific rabbit antiserum (27) at a dilution of 1:10,000, while PrV gD was detected using monospecific rabbit antiserum (16/00) (16) at a dilution of 1:20,000. Peroxidase-conjugated goat anti-mouse or goat anti-rabbit secondary antibodies (Jackson ImmunoResearch Laboratories Inc.) were used at a dilution of 1:20,000.

### Statistical analysis.

Statistical analyses and graphical presentations were computed with GraphPad Prism 8 software. Two-tailed Student’s *t* test with Welch’s correction was used to assess significant differences. *N* represents the number of independent experiments. *P* values of ≤0.05 or less were considered statistically significant.
