# Supplementary figures and images for: In Vitro Viral Evolution Identifies a Critical Residue in the Alphaherpesvirus Fusion Glycoprotein B Ectodomain That Controls gH/gL-Independent Entry
Source: mBio. 2021 May 4;12(3):e00557-21. doi: 10.1128/mBio.00557-21 (PMC8262866; doi:10.1128/mBio.00557-21)

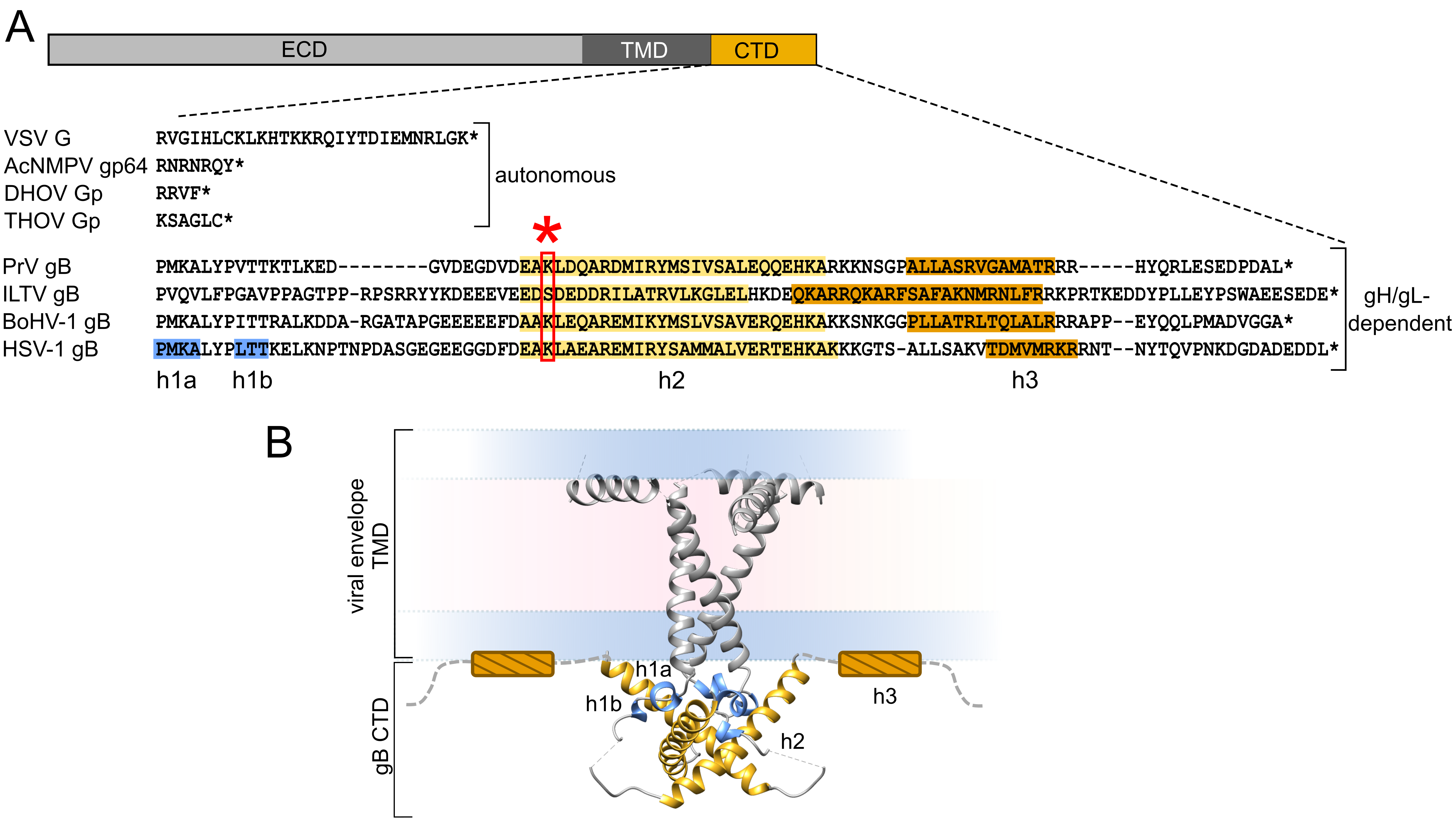

Supplement: FIG S1 [file mbio.00557-21-sf001.tif]

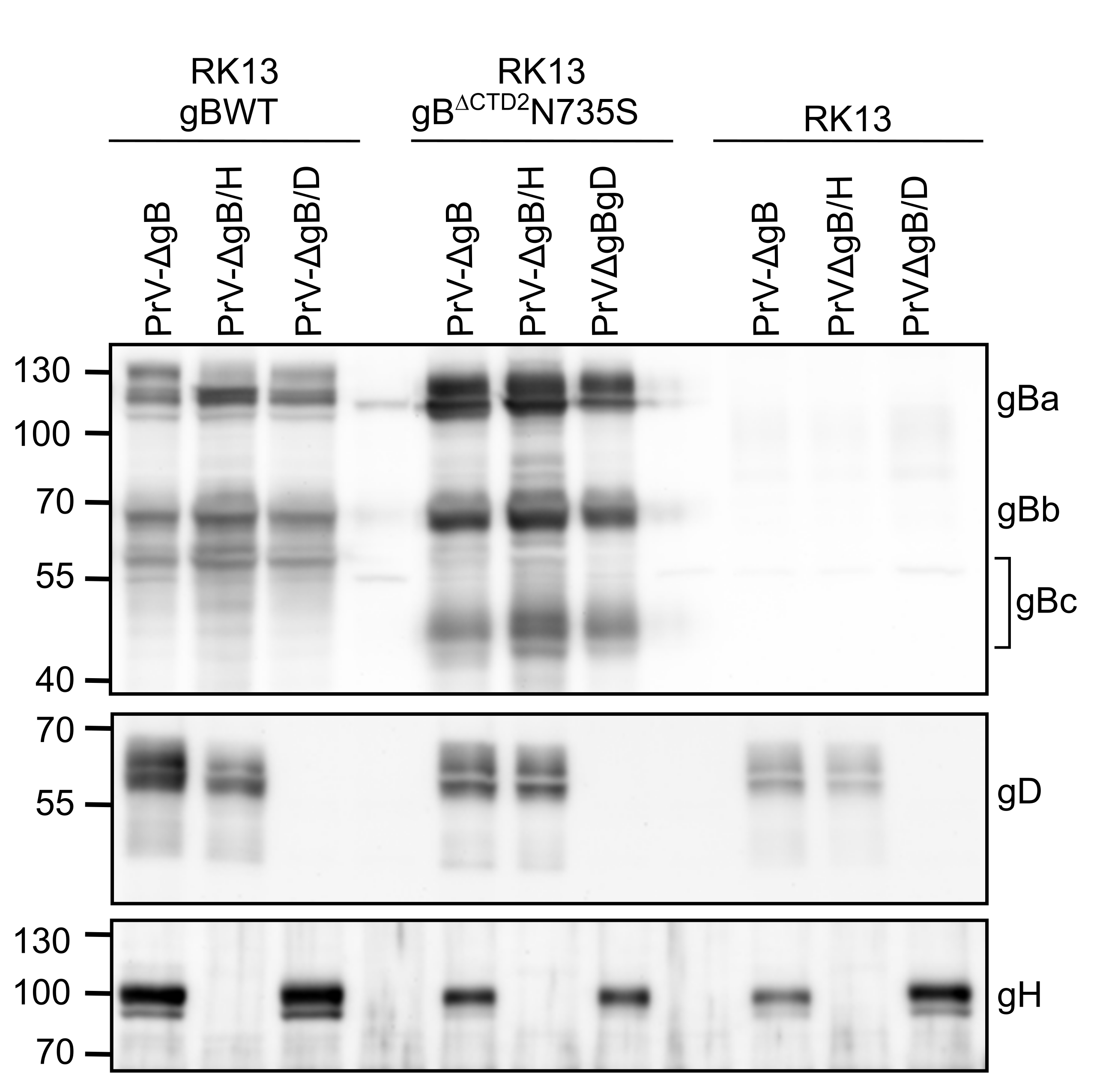

Supplement: FIG S2 [file mbio.00557-21-sf002.tif]

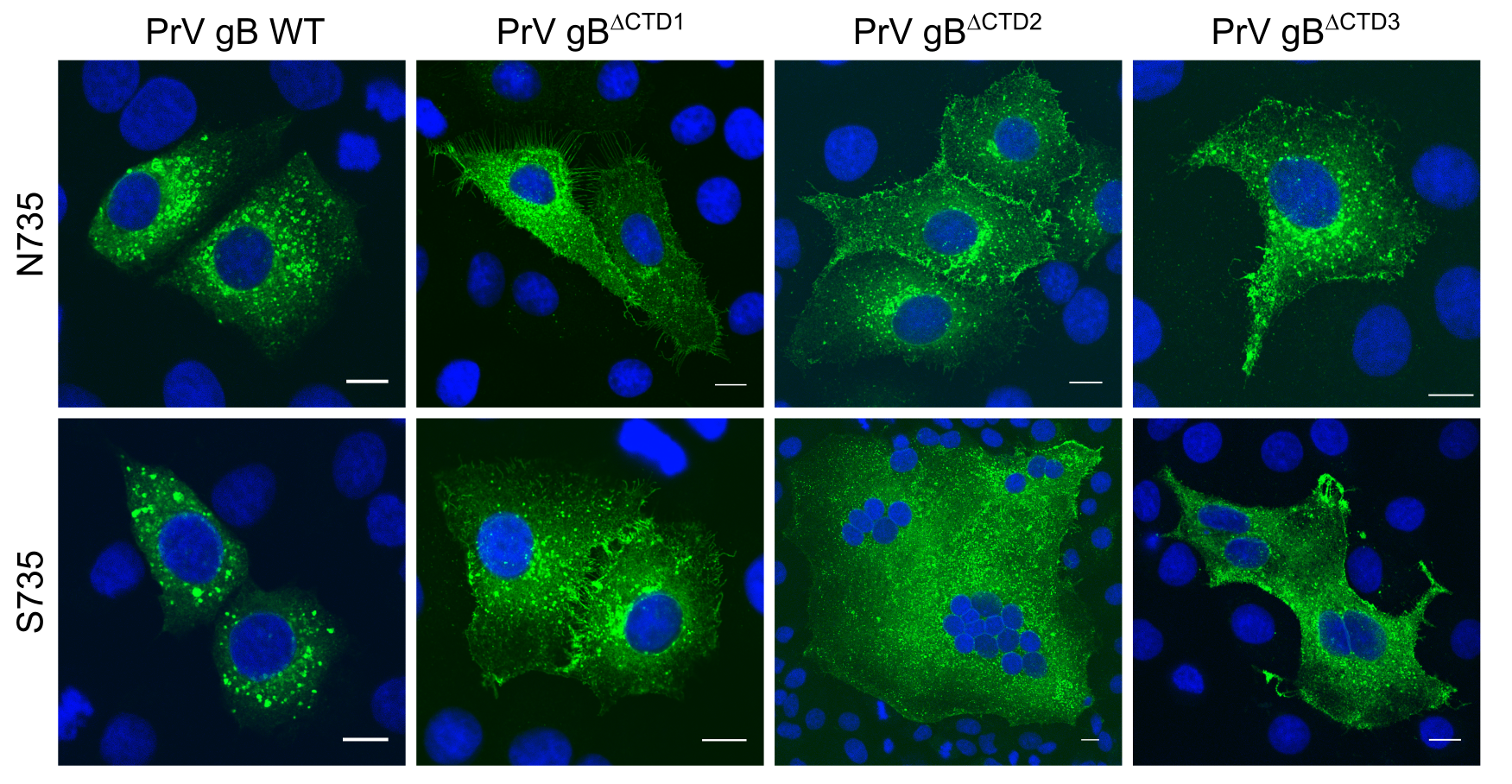

Supplement: FIG S3 [file mbio.00557-21-sf003.tif]

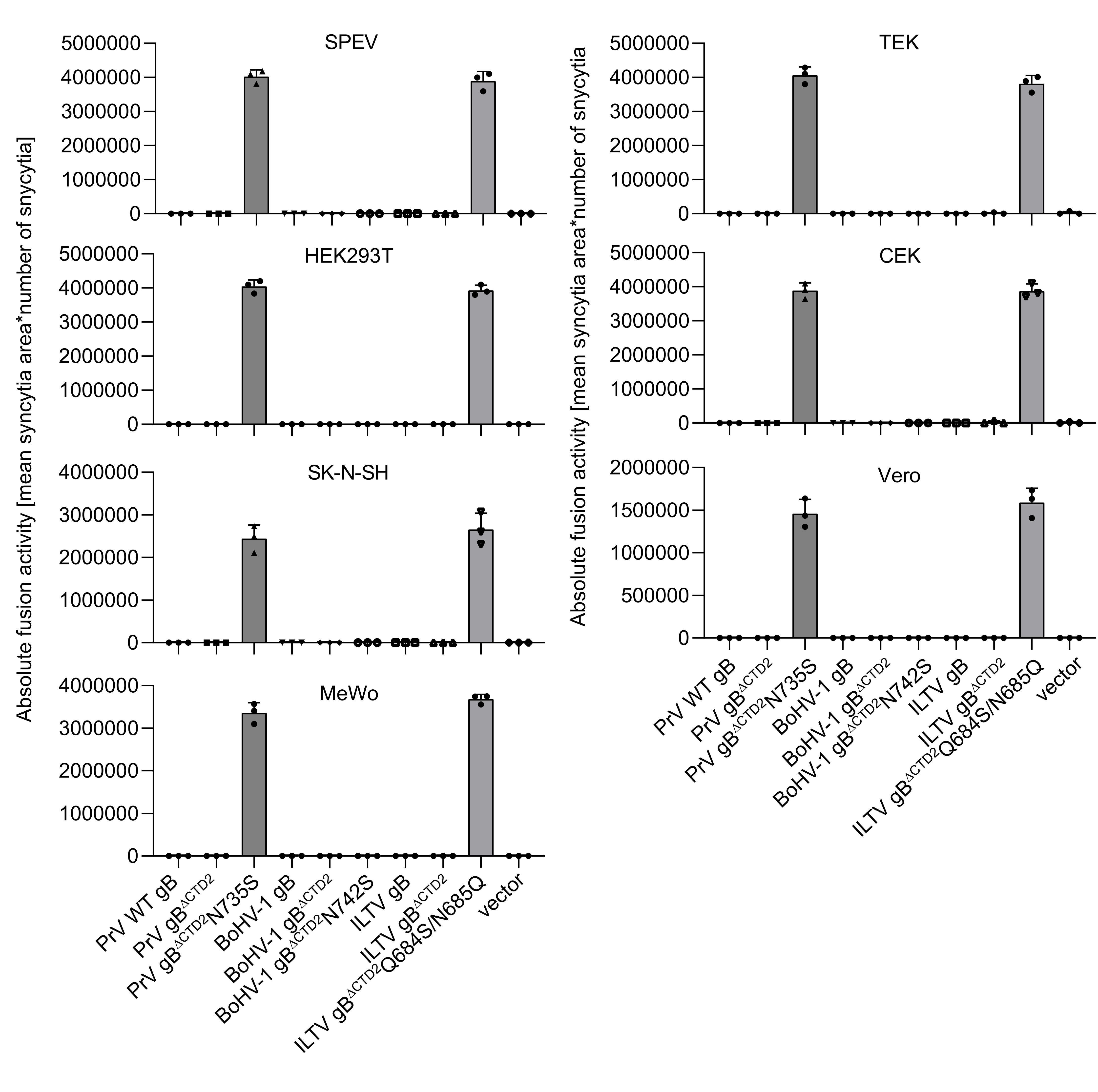

Supplement: FIG S4 [file mbio.00557-21-sf004.tif]
