# Supplementary material for: In Vitro Viral Evolution Identifies a Critical Residue in the Alphaherpesvirus Fusion Glycoprotein B Ectodomain That Controls gH/gL-Independent Entry
Source: mBio. 2021 May 4;12(3):e00557-21. doi: 10.1128/mBio.00557-21 (PMC8262866; doi:10.1128/mBio.00557-21)
Supplement: TABLE S1 [file mbio.00557-21-st001.docx]

**Table S1.**

Primers used in this study. Non matching nucleotides are in italics, restriction sites used for cloning are underlined and mutations introduced by site-directed mutagenesis are in bold.

| **Gene** | **Primer name** | **Sequence (5´à 3´)** |
| --- | --- | --- |
| PrV gB | gB-Ass_for | *TAACGG*ATCCATGCCCGCTGGTGGCGG |
|  | GBC-37_rev | *CCGAATTC*CTAGGCCTCGTCCACGTCGCCTTC |
|  | PrV-gBPro_for | *CACA*GGATCCTGGCGCGCTTCATGG |
|  | gB-Ass_rev | *CAGAATTC*CTACAGGGCGTCGGGGTCC |
|  | 130 | CGTGCCCGTCCCCGTGCAGGAGATC |
|  | 134 | CCATCTACCGGCGGCGCTACAACA |
|  | 144 | AGCGCCAGCAGCAGCAGCGCTAG |
|  | P16 | CGGTGCTGGCCTCGGACGTCT |
|  | PrVgB-N735S-F | GATCCAGCGCCGC***AG***CCAGCTGCACGCGC |
|  | PrVgB-N735S-R | GCGCGTGCAGCTGG***CT***GCGGCGCTGGATC |
|  | PrVgB-LL890/891AA-F | GGCCCGCG***GC***G***GC***GGCCAGCCGCGTC |
|  | PrVgB-LL890/891AA-R | GACGCGGCTGGCC***GC***C***GC***CGCGGGCC |
|  | PrVgB-Y905A-F | CGCCGCCGGCACGCCCAGCGCCTCGAGAG |
|  | PrVgB-Y904A-R | CTCTCGAGGCGCTGGGCGTGCCGGCGGCG |
| PrV gH | DH3 | *AGAATTC*AAAGTTTGCCGTGCCCGTC |
|  | DH1 rev | *CATCTAGA*CACGCGCACGCAGAGAGT |
|  | WH3 | TGCACGAGAGCGACGACTACC |
| PrV gL | UL1 for | *CACAAAGCTT*AGGATACACCAGCCGCGATG |
|  | UL1 rev | *CACAGAATTC*GGTCTCTTACTCGGCGGGGG |
| PrV gD | US6_for | *CACAGAATTC*ACCTGCCAGCGCCATGC |
|  | US6_rev | *CACAGAATTC*CATCGACGCCGGTACTGC |
| BoHV-1 gB | BoHV-1-gB_for | *CACAGGATCC*ATGGCCGCTCGCGGC |
|  | BoHV-1-gB_rev | *CACAGAATTC*TCATGCCCCCCCGAC |
|  | BoHV-1-gB^∆CTD2^EcoRI | *CACAGAATTC*CTAGGCCGCGTCAAACTCC |
|  | BoHV-1-gBN742S_F | GATACAGCGCCGC***AG***CCAGCTGCACGAGC |
|  | BoHV-1-gBN742S_R | GCTCGTGCAGCTGG***CT***GCGGCGCTGTATC |
|  | BoHV-1-gB_seq_T277 | CACGGGCACCTCTGTGAACTGCATC |
| BoHV-1 gH | BHV-gH_for | *CACAGAATTC*ATGCGGCGCCCGCTC |
|  | BHV-gH_rev | *CACATCTAGA*CTAAAACACCGGGAC |
| BoHV-1 gL | BHV-gL_for | *CACAGGATCC*ATGGCACCGGCGGTC |
|  | BHV-gL_rev | *CACAGAATTC*CTAGCGGTAGATGCC |
| BoHV-1 gD | BHV-gD_for | *CACAGGATCC*ATGCAAGGGCCGACA |
|  | BHV-gD_rev | *CACAGAATTC*TC ACCCGGGCAGCGC |
| ILTV gB | IgB-F1 | *ACGGATCC*AAAGATTTATACGCGGTCCTG |
|  | IgB-R1 | *CAGAATTC*ACTTATTCGTCTTCGCTTTCTTC |
|  | IgBtrunc-F | GAGGAGGAGGTTGAGGAGGAT***T***G***AAT***T***C***AGGACGACAGGATACTTGCC |
|  | IgBtrunc-R | GGCAAGTATCCTGTCGTCCT***G***A***ATT***C***A***ATCCTCCTCAACCTCCTCCTC |
|  | IgBSQ-F | GGACTTTAAACTATGATGATGTGGTCAGATAT***TC***A***C***A***G***ATTTATAACAAAAGGTTCAGAGAC |
|  | IgBSQ-R | GTCTCTGAACCTTTTGTTATAAAT***C***T***G***T***GA***ATATCTGACCACATCATCATAGTTTAAAGTCC |
| HSV-1 gB | HSV gB N709S-F | GGTCCAGCGCCGCA**G**CCAGCTGCACGACC |
|  | HSV gB N709S-R | GGTCGTGCAGCTGG**C**TGCGGCGCTGGACC |
|  | HSV-1-gB-F | *CACA*GGATCCGTCCCGCC*ATG*CGCCAG |
|  | HSV-1-gBK839* | GG*CAATTCCTAG*GCCTCGTCAAAGTCGCCGCC |
|  | HSV-1-gBA317 | GCCAAGGGGGTCTGTCGGTCC |
|  | HSV-1-gBR481 | CGCGACGCCATGGACCGCATC |
| pcDNA3 | T7 | TAATACGACTCACTATAGGG |
|  | SP6 | CTCTAGCATTTAGGTGACACTATAG |
